# Isovaleryl Sucrose Esters from *Atractylodes japonica* and Their Cytotoxic Activity

**DOI:** 10.3390/molecules29133069

**Published:** 2024-06-27

**Authors:** Yimeng Wang, Zhibin Wang, Yanping Sun, Mingtao Zhu, Yong Jiang, Haodong Bai, Bingyou Yang, Haixue Kuang

**Affiliations:** Key Laboratory of Basic and Application Research of Beiyao, Ministry of Education, Heilongjiang University of Chinese Medicine, Harbin 150040, China; yimengwang6642@163.com (Y.W.); wzbmailbox@163.com (Z.W.); sunyanping_1@163.com (Y.S.); zmingt180@163.com (M.Z.); jy1282824472@163.com (Y.J.); bhd2032@163.com (H.B.); ybywater@163.com (B.Y.)

**Keywords:** phytochemistry, isovaleryl sucrose esters, cytotoxic activity, molecular docking, molecular dynamics simulation

## Abstract

Cancer represents one of the most significant health challenges currently facing humanity, and plant-derived antitumour drugs represent a prominent class of anticancer medications in clinical practice. Isovaleryl sucrose esters, which are natural constituents, have been identified as having potential antitumour effects. However, the mechanism of action remains unclear. In this study, 12 isovaleryl sucrose ester components, including five new (**1**–**5**) and seven known compounds (**6**–**12**), were isolated from the roots of *Atractylodes japonica.* The structures of the compounds were elucidated using 1D and 2D-NMR spectroscopy, complemented by HR-ESI-MS mass spectrometry. The cytotoxic activities of all the compounds against human colon cancer cells (HCT-116) and human lung adenocarcinoma cells (A549) were also evaluated using the CCK8 assay. The results demonstrated that compounds **2**, **4**, and **6** were moderately inhibitory to HCT-116 cells, with IC_50_ values of 7.49 ± 0.48, 9.03 ± 0.21, and 13.49 ± 1.45 μM, respectively. Compounds **1** and **6** were moderately inhibitory to A549, with IC_50_ values of 8.36 ± 0.77 and 7.10 ± 0.52 μM, respectively. Molecular docking revealed that compounds **1**–**9** exhibited a stronger affinity for FGFR3 and BRAF, with binding energies below −7 kcal/mol. Compound **2** exhibited the lowest binding energy of −10.63 kcal/mol to FGFR3. We screened the compounds with lower binding energies, and the protein-ligand complexes already obtained after molecular docking were subjected to exhaustive molecular dynamics simulation experiments, which simulated the dynamic behaviour of the molecules in close proximity to the actual biological environment, thus providing a deeper understanding of their functions and interaction mechanisms. The present study provides a reference for the development and use of iso-valeryl sucrose esters in the antitumour field.

## 1. Introduction

Cancer is one of the most significant health challenges currently facing humanity [1], and plant-derived antitumour drugs represent a prominent class of anticancer medications in clinical practice [2]. Examples of such drugs include the topoisomerase inhibitor camptothecin [3], the antimitotic tumour drug paclitaxel [4], and the tumour differentiation and apoptosis-inducing elemene [5]. Consequently, the discovery and isolation of targeted antitumour drugs from traditional Chinese medicine with the potential for mass production, high efficiency, and low toxicity has been a subject of intense research interest for numerous scientists.

*Atractylodes japonica*, a member of the genus *Atractylodes* DC belonging to the family Asteraceae, is a native medicinal herb in northeast China and is mainly distributed in the Jilin, Heilongjiang, and northeast Liaoning Provinces [6]. This herb is widely used in northeast China for the treatment of ailments such as spleen pain, abdominal pain, diarrhoea, lethargy, and weakness [7]. According to the existing literature [8], *A. japonica* contains a type of sucrose ester with only isovaleryl as a substituent, which is rare in natural products. Sucrose esters are non-ionic surfactants often produced in the laboratory from sucrose and fatty acids by esterification. They exhibit a range of biological activities, including antibacterial, anti-inflammatory, antitumour, and insecticidal activities [9,10]. Sucrose esters with only isovaleryl as a substituent are relatively rare among natural products, with only 25 compounds reported to date. Of these, 21 are from the Asteraceae family, including seven from *A. lancea* [11,12], four from *A. japonica* [8] and eight from *A. yunnanensis*. Among these, ainsloside B showed remarkable cytotoxicity against A549 lung cancer cells with an IC_50_ value of 3.3 μM [13]. In addition, two compounds have been identified in *Chrysanthemum maculatum* [14]. Only four isovaleryl sucrose esters have been reported from other families. Three of these are from the roots of *Brassica rapa* ssp. campestris of the Brassicaceae family [15], while the fourth is from Euphorbiae Lathyridis Semen of the Euphorbiaceae family [16], which has been shown to inhibit melanogenesis. It can be observed that these ingredients exhibit cytotoxic activity against a range of tumour cells. However, there is a paucity of literature on the mechanism of action of these compounds. The BRAF and FGFR genes are aberrantly expressed in a variety of cancers, as evidenced by studies [17,18]. According to literature reports, aberrant expression of BRAF is present in 4.7–22.6% of patients with colorectal cancer [19,20]; Aberrant expression of FGFR3 can lead to the development and progression of lung cancer, particularly non-small cell lung cancer [21,22]. Moreover, BRAF inhibitors [23,24] and FGFR inhibitors [25,26,27] are currently the subject of intense research, with some of these inhibitors already being applied in clinical practice. Therefore, these two proteins were the focus of this study.

Therefore, the aim of this study was to isolate and characterise isovaleryl sucrose esters from *Atractylodes japonica* and to investigate their cytotoxic activity. To explore the mechanism of action of all compounds, we subjected all the compounds to molecular docking and molecular dynamics simulations with FGFR3 and BRAF proteins, respectively. 

**Figure 1:**
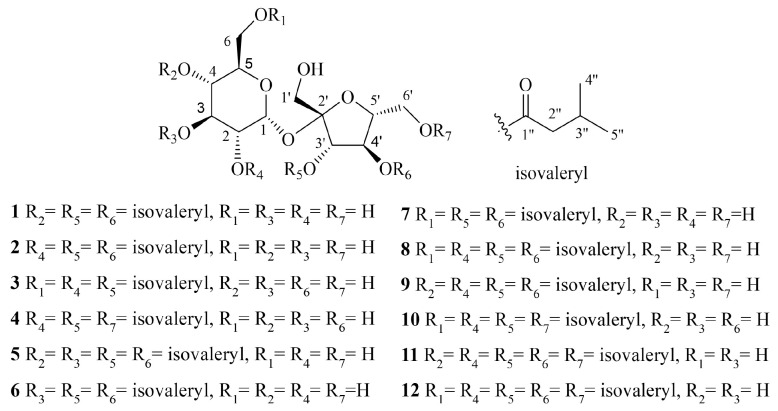
Structures of compounds **1**–**12**.

In this study, 12 isovaleryl sucrose esters were isolated from *A. japonica* (Figure 1), including five new isovaleryl sucrose esters (**1**–**5**) and seven known isovaleryl sucrose esters (**6**–**12**). The cytotoxic activities of all the compounds against colon cancer cells HCT-116 and lung adenocarcinoma cells A549 were evaluated using the Cell Counting Kit-8 (CCK-8) assay. The results showed that compounds **2**, **4**, and **6** were moderately inhibitory to HCT-116 cells, with IC_50_ values of 7.49 ± 0.48, 9.03 ± 0.21, and 13.49 ± 1.45 μM, respectively. Compounds **1** and **6** were moderately inhibitory to A549, with IC_50_ values of 8.36 ± 0.77 and 7.10 ± 0.52 μM, respectively. The results show that compounds **1**–**10** have lower binding energies to these two proteins, all below −7 kcal/mol. We carried out molecular dynamics simulation experiments with compounds **2**, **4**, **6**–**8**, which have lower binding energies, and the results show that the binding of these compounds to the proteins is relatively stable. The results suggest that such compounds may exert cytotoxic activity against HCT-116 and A549 cells by acting on FGFR3 and BRAF proteins. Because these compounds are easy to synthesise in the laboratory, they have some development potential.

## 2. Results

### 2.1. Identification of the Isolated Compounds

Compound **1**, characterised as a yellow oil, ${\left[\alpha \right]}_{D}^{25}$: 6.469° (c 0.17, CH_3_OH). Its molecular formula was determined to be C_27_H_46_O_14_, as evidenced by its HR-ESI-MS data (*m*/*z* 617.2787 [M + Na]^+^, calcd. for 617.2780) (Figure S1). The IR spectra indicate the presence of hydroxyl (3434 cm^−1^) and carbonyl groups (1734 cm^−1^) (Figure S2). The ^13^C-NMR (150 MHz, CDCl_3_) spectrum of compound **1** (Table 1, Figure S4) revealed the presence of a total of 27 carbon signals. These signals can be attributed to six methyl groups at *δ*_C_ 22.6 (C-4″/5″) × 2, 22.5 (C-4″/5″), and 22.4 ppm (C-4″/5″) × 3. Three methylene groups were identified at *δ*_C_ 43.5 (C-3″) and 43.1 ppm (C-3″) × 2, along with Three hypomethyls were observed at *δ*_C_ 25.9 (C-2″) and 25.7 ppm (C-2″) × 2.

Additionally, the spectra indicated the presence of a group of sucrose signals [28] at δ_C_ 92.0 (C-1), *δ*_C_ 72.1 (C-2), *δ*_C_ 71.9 (C-3), and *δ*_C_ 70.3 (C-4). *δ*_C_ 72.2 (C-5), *δ*_C_ 61.2 (C-6), *δ*_C_ 61.1 (C-1′), *δ*_C_ 104.5 (C-2′), *δ*_C_ 77.7 (C-3′), *δ*_C_ 74.2 (C-4′), *δ*_C_ 82.0 (C-5′), and *δ*_C_ 64.9 ppm (C-6′). It is noteworthy that the downfield signals, specifically at *δ*_C_ 173.7 (C-1″), 172.6 (C-1″), and 172.3 ppm (C-1″), confirm the ester carbonyl resonances. In the ^1^H-NMR (600 MHz, CDCl_3_) spectrum (Table 2, Figure S3), the signal at *δ*_H_ 5.53 ppm (1H, d, *J* = 3.5) is the anomeric proton signal for glucose, which is indicative of α-glucose due to its small coupling constant. 

**Figure 2:**
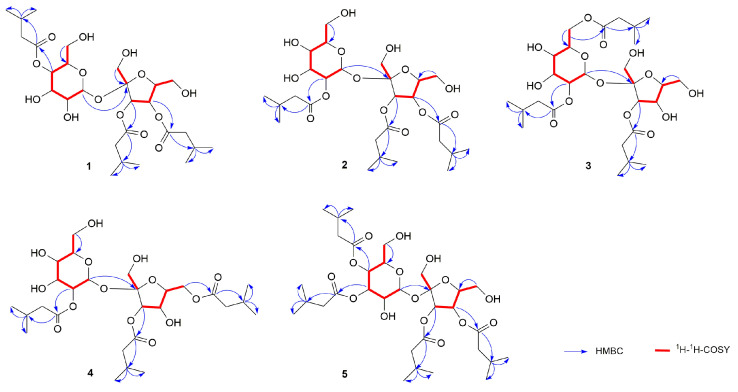
Key HMBC and ^1^H, ^1^H-COSY correlation of compounds **1**–**5**.

**Figure 3:**
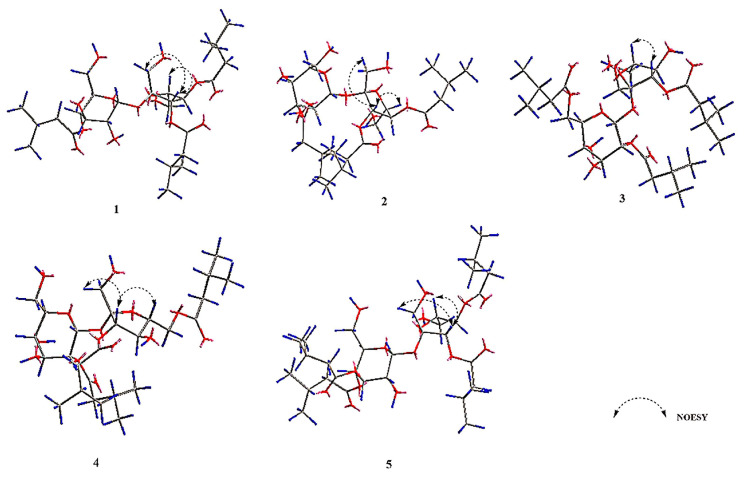
Key NOESY of compounds **1**–**5** (Dark grey represents carbon atoms, blue represents hydrogen atoms and red represents oxygen atoms in the diagram).

In ^1^H-^1^H COSY (Figure S8), we observed correlations for H-2″/H-3″ and H-4″/H-5″. Additionally, in HMBC (Figure S8), correlations for C-1″/H-2″ were noted. Based on these findings, the aglycone can be identified as having three isovaleryl moieties. In the Nuclear Overhauser Effect Spectroscopy (NOESY) analysis (Figure S9), we observed correlations of H-1′/H-3′ and H-3′/H-5′. These findings suggest the relative conformation of fructose to be *β*-fructose. In the HMBC data (Figure S8), the observed correlations of H-1/C-2′ indicate that the C-1 position of α-glucose is linked to the C-2′ position of *β*-fructose. Upon subsequent acid hydrolysis, the absolute configuration of α-glucose and *β*-fructose was conclusively determined to be D-type [29]. In summary, the compound possesses one sucrose moiety and three isovaleryl moieties. Compared to the ^13^C-NMR of sucrose [28], the chemical shifts of C-4, C-3′, and C-4′ in compound **1** exhibit downfield movement, while the adjacent carbon chemical shifts show upfield movement. This implies that the sucroses C-4, C-3′, and C-4′ might be substituted by isovaleryl moieties. In HMBC (Figure S8), *δ*_C_ 173.7 (C-1″), 172.6 (C-1″), and 172.3 ppm (C-1″) display correlations with *δ*_H_ 4.86 (1H, t, *J* = 9.6, H-4), 5.37 (1H, d, *J* = 5.9, H-3′), and 5.43 ppm (1H, t, *J* = 5.9, H-4′), respectively. This data suggests that the aglycones connect to the C-4, C-3′, and C-4′ positions of sucrose. Consequently, the compound is identified as 4,3′,4′-triisovaleryl-*β*-d-fructofuranosyl-*α*-d-glucopyranoside (Figure 1, Figure 2 and Figure 3).

Compounds **2** to **4** have the same molecular weight as compound **1** (Figures S10, S19 and S28). The IR spectra of compounds **2** to **4** also display the presence of carbonyl and hydroxyl groups (Figures S2, S11 and S20). Comparing the ^1^H-NMR (Figures S3, S12 and S21) and ^13^C-NMR (Figures S4, S13 and S22) of compounds **2** to **4**, we found that both compounds have the same parent nucleus and substituents as compound **1**. The distinction is in the substitution position of the isovaleryl groups on sucrose (Figure 1, Figure 2 and Figure 3). 

Compound **2** is a yellow oil, ${\left[\alpha \right]}_{D}^{25}$: 6.614° (c 0.26, CH_3_OH). In the HMBC spectrum (Figure S17), *δ*_C_ 173.4 (C-1″), 172.6 (C-1″), and 172.5 ppm (C-1″) show correlations with *δ*_H_ 4.77 (dd, *J* = 10.1, 3.7, H-2), 5.48 (d, *J* = 7.4, H-3′), and 5.45 ppm (t, *J* = 7.1, H-4′), respectively. Based on this, compound **2** was identified as 2,3′,4′-triisovaleryl-*β*-d-fructofuranosyl-α-d-glucopyranoside (Figure 1, Figure 2 and Figure 3 and Table 1 and Table 2).

Compound **3**, ${\left[\alpha \right]}_{D}^{25}$:7.064° (*c* 0.26, CH_3_OH) is a yellow oil. In the HMBC spectrum (Figure S21), three carbonyl carbon signals at *δ*_C_ 174.7 (C-1″), 174.2 (C-1″), and 174.1 ppm (C-1″) correlate with *δ*_H_ 4.63 ppm (1H, dd, *J* = 10.1, 3.8, H-2), 4.45 (1H, dd, *J* = 12.0, 2.0, H-6a), 4.16 (1H, dd, *J* = 12.0, 5.3, H-6b) and 5.43 ppm (1H, d, *J* = 8.6, H-3′). This suggests that the three isovaleryl groups are attached at positions C-2, C-6, and C-3′ of sucrose. Thus, compound **3** was identified as 2,6,3′-triisovaleryl-*β*-d-fructofuranosyl-*α*-d-glucopyranoside (Figure 1, Figure 2 and Figure 3 and Table 1 and Table 2). 

Compound **4** is a yellow oil, ${\left[\alpha \right]}_{D}^{25}$: 7.640° (*c* 0.26, CH_3_OH). In the HMBC spectrum (Figure S26), *δ*_C_ 174.7 (C-1′’), 174.1 (C-1′’), and 174.0 (C-1′’) show correlations with 4.62 (1H, dd, *J* = 10.2, 3.8, H-2), 5.45 (1H, d, *J* = 8.6, H-3′), and 4.35 (2H, m, H-6), respectively. Based on this, compound **4** was identified as 2,3′,6′-triisovaleryl-*β*-d-fructofuranosyl-*α*-d-glucopyranoside (Figure 1, Figure 2 and Figure 3 and Table 1 and Table 2).

Compound **5** was isolated as a yellow oil, ${\left[\alpha \right]}_{D}^{25}$: +9.270° (*c* 0.27, CH_3_OH). Its molecular formula, C_32_H_54_O_15_, was confirmed based on HR-ESI-MS data (*m*/*z* 701.3371 [M + Na]^+^, clcd. for 701.3355) (Figure S36). The IR spectrum (Figure S37) revealed the presence of hydroxyl (3422 cm^−1^) and carbonyl (1734 cm^−1^) groups. The structure of compound **5** is similar to that of compound **1**, except that compound **5** is replaced by four isovaleryl groups. In the HMBC spectrum (Figure S44), correlations between these carbonyl carbons and 5.32 (1H, t, *J* = 9.8, H-3), 5.08 (1H, t, *J* = 9.8, H-4), 5.64 (1H, d, *J* = 7.2, H-3′), and 5.48 (1H, t, *J* = 7.3, H-4′) confirmed that the 4 isovaleryl groups were attached to the C-3, C-4, C-3′, and C-4′ of the sucrose unit, respectively. Consequently, compound **5** was identified as 3,4,3′,4′-tetrakis-isovaleryl-*β*-d-fructofuranosyl-*α*-d-glucopyranoside (Figure 1, Figure 2 and Figure 3 and Table 1 and Table 2).

The seven known compounds **6**–**12** were respectively identified as 3,3′,4′-triisovalerate-*β*-d-fructofuranosyl-α-d-glucopyranoside (**6**) [13], 6,3′,4′-triisovalerate-*β*-d-fructofuranosyl-α-d-glucopyranoside (**7**) [13], 2,6,3′,4′-tetrakis-isovaleryl-*β*-d-fructofuranosyl-α-d-glucopyranoside (**8**) [9], 2,4,3′,4′-tetrakis-isovaleryl-*β*-d-fructofuranosyl-α-d-glucopyranoside (**9**) [9], 2,6,3′,6′-pentaisovalerate-*β*-d-fructofuranosyl-α-d-glucopyranoside (**10**) [9], 2,4,3′,4′,6′-pentaisovalerate-*β*-d-fructofuranosyl-α-d-glucopyranoside (**11**) [10], 2,6,3′,4′,6′-pentaisovalerate-*β*-d-fructofuranosyl-α-d-glucopyranoside (**12**) [10] (Tables S1 and S2). 

### 2.2. Cytotoxicity Assays

The results demonstrated that compounds **2**, **4**, and **6** exhibited moderate inhibitory activity against HCT-116 cells, with IC_50_ values of 7.49 ± 0.48, 9.03 ± 0.21, and 13.49 ± 1.45 μM. The inhibitory effect of compounds **1** and **6** against A549 was found to be moderate, with IC_50_ values of 8.36 ± 0.77 and 7.10 ± 0.52 μM, respectively (Table 3).

### 2.3. Docking Study

**Figure 4:**
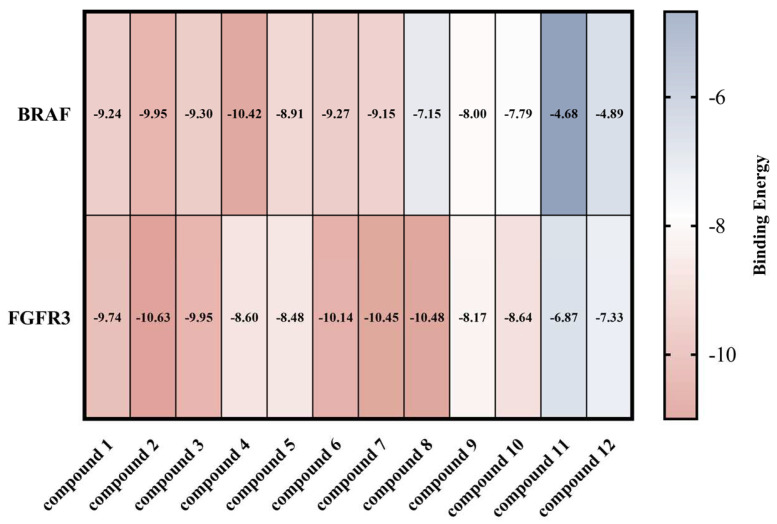
Binding energy (kcal/mol) heat map of compounds **1**–**12** docked to BRAF and FGFR3 proteins, respectively.

**Figure 5:**
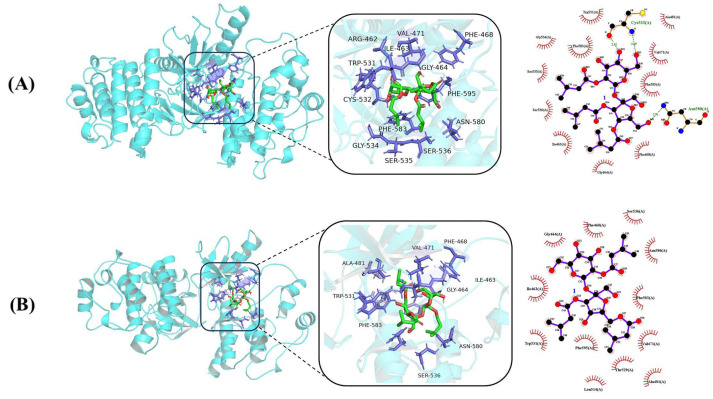

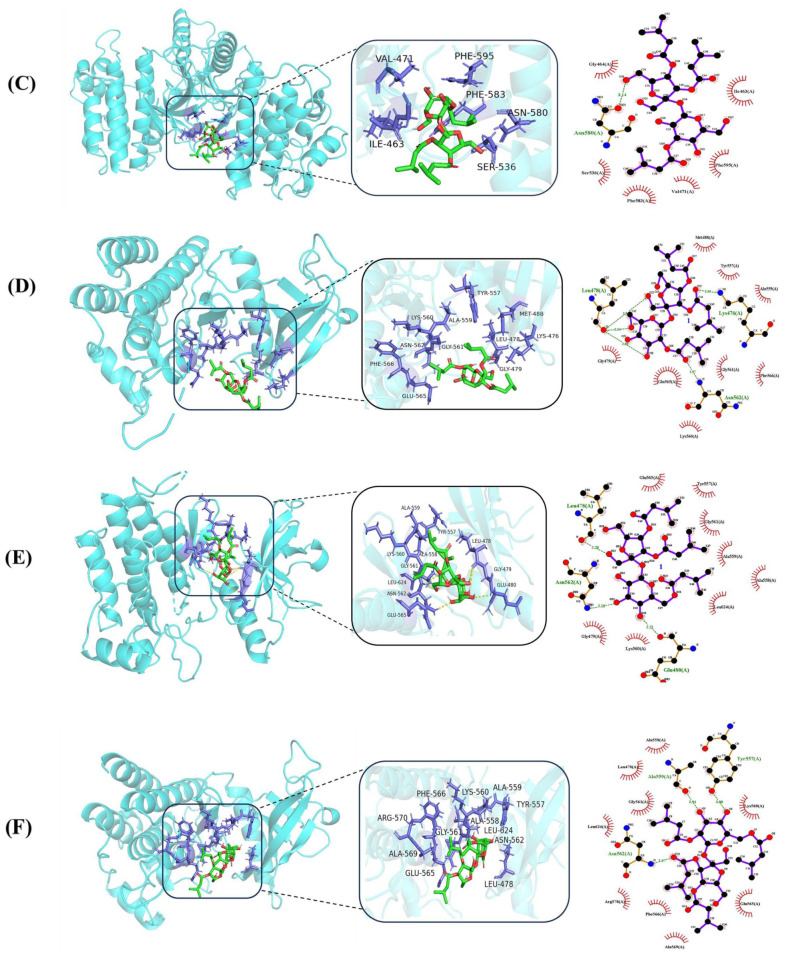
Visualisation results of molecular docking of compounds to proteins: (**A**) is compound **2** with BRAF (binding energy −9.95 kcal/mol), (**B**) is compound **4** with BRAF (binding energy −10.42 kcal/mol), (**C**) is compound **6** with BRAF (binding energy −9.27 kcal/mol), (**D**) is compound **2** with FGFR3 (binding energy −10.63 kcal/mol), (**E**) is compound **7** with FGFR3 (binding energy −10.45 kcal/mol), and (**F**) is compound **8** with FGFR3 (binding energy −10.48 kcal/mol). (In the three-dimensional diagram, green represents small molecule ligands, blue represents receptor proteins, and purple represents amino acid residues interacting with the ligand. In the two-dimensional diagram, purple represents ligands and red represents amino acid residues interacting with the ligand).

Molecular docking is a technique for predicting the binding of ligands to proteins with known three-dimensional structures [30]. All compounds were subjected to molecular docking experiments with FGFR3 (PDBID: 6LVM) and BRAF (PDBID: 8C7X), respectively. The binding energies of compounds **1**–**10** in pairs with BRAF and FGFR3 were lower than −7 kcal/mol (Figure 4), indicating better binding. The original ligand for BRAF (PDBID: 8C7X) is *N*-(3-(5-chloro-1*H*-pyrrolo[2,3-b] pyridine-3-carbonyl)-2,4-difluorophenyl)-3-(2-cyanopropan-2-yl) benzamide [31], with a binding energy of −13.00 kcal/mol. The compounds with the lowest binding energies to BRAF among our compounds are compound **2**, **4**, and **6**, with binding energies of −9.95, −10.42, and −9.27 kcal/mol, respectively (Figure 4). A visual analysis of the docking results conducted using LigPlot 2.2.8 and PyMol 2.5.5 revealed that these compounds were hydrogen and non-hydrogen bonded to the Cys532, Asn580, Trp531, Ala481, Val471, Phe595, Phe468, Gly464, Ile463, Ser 536, Ser535, Gly534, Phe583, Thr529, Leu 514, Trp 595 and other sites (Figure 4 and Figure 5A–C). The original ligand for FGFR3 (PDBID: 6LVM) is 2-[(5-[2-(3,5-Dimethoxyphenyl)ethyl]-2-{3-methoxy-4-[4-(4-methylpiperazin-1-yl)piperidin-1-yl]anilino}pyrimidin-4-yl)amino]-*N*-ethylbenzene-1-sulfonamide is a novel FGFR3 inhibitor [32], which has a binding energy of −9.06 kcal/mol. In contrast, compounds **2**, **7**, and **8** had the lowest binding energies to FGFR3 with −10.63, −10.45, and −10.48 kcal/mol, respectively (Figure 4). The components in question exhibit either hydrogen bonding or non-hydrogen bonding at Leu478, Lys476, Met 488, Tyr557, Ala559, Phe 566, Gly561, Glu565, Gly479, Glu480, Gly557, Ala558, Leu624, Lys560, Asn562, Ala569, Phe566, Arg570, and other sites of the FGFR3 protein (Figure 4 and Figure 5D–F). As illustrated in Figure 4, these compounds demonstrate enhanced connectivity with the target and augmented binding efficacy, thereby corroborating the cytotoxic activity of compounds **1**–**12**.

### 2.4. Molecular Dynamics Simulation

#### 2.4.1. Stability Analysis of Small Molecule-Protein Receptor Complexes

Root-mean-square deviation (RMSD) [33] curves are of significant importance in molecular dynamics simulations, serving as a crucial indicator for the assessment of structural stability in protein-ligand complexes. 

**Figure 6:**
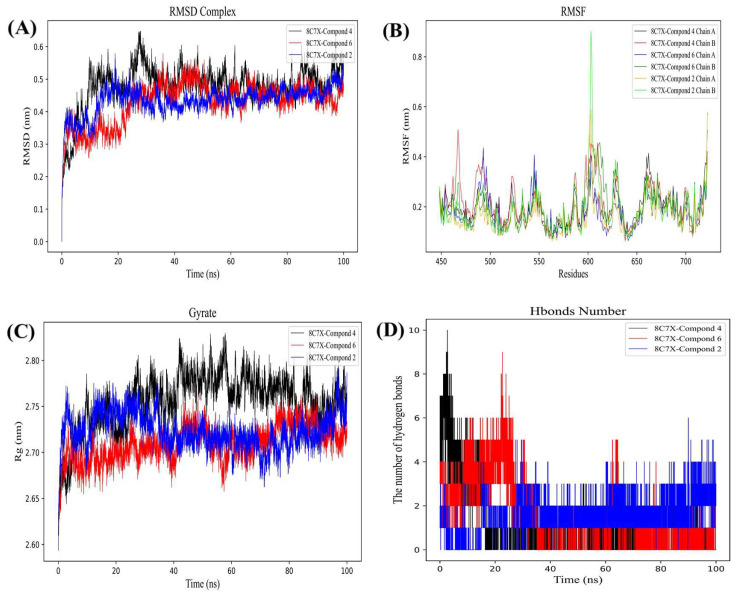
Molecular dynamics simulation results: graph (**A**) RMSD of compounds **2**, **4,** and **6** with BRAF. (**B**) RMSF of compounds **2**, **4**, and **6** with BRAF. (**C**) Rg of compounds **2**, **4**, and **6** with BRAF. (**D**) H bond number of compounds **2**, **4**, and **6** with BRAF.

**Figure 7:**
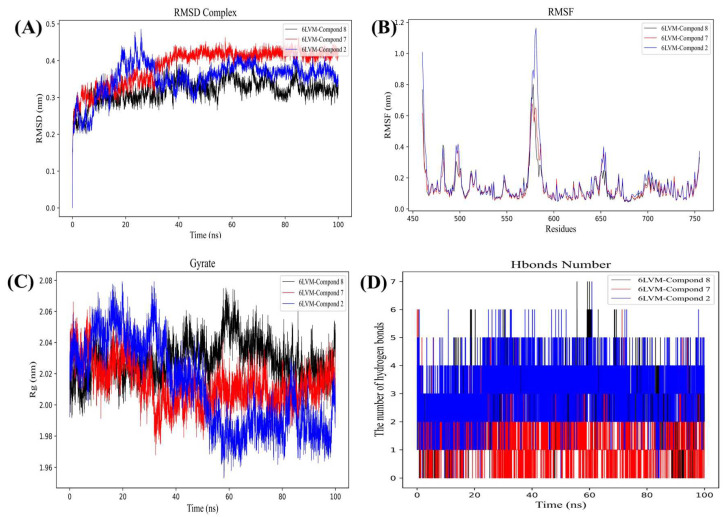
Molecular dynamics simulation results: graph (**A**) is RMSD of compounds **2**, **7** and **8** with FGFR3. (**B**) RMSF of compounds **2**, **7**, and **8** with FGFR3. (**C**) Rg of compounds **2**, **7**, and **8** with FGFR3. (**D**) H bond number of compounds **2**, **7**, and **8** with FGFR3.

As illustrated in Figure 6A, the RMSD curve of compound **2**-BRAF (blue line) remains relatively stable at approximately 0.45 nm for a duration of 15–100 ns. The RMSD curve of compound **4**-BRAF (black line) is stable around 0.45–0.50 nm for 35–100 ns, and the RMSD curve of compound **6**-BRAF (red line) indicates that the compound is relatively stable at 0.45–0.50 nm for 35–100 ns, which suggests that compounds **2**, **4,** and **6** can form a stable complex system with BRAF. As shown in Figure 7A, the RMSD curve of compound **2**-FGFR3 (blue line) is stable around 0.33 nm for 30–100 ns. The RMSD curve of compound **7**-FGFR3 (red line) is relatively stable around 0.36–0.40 nm for 60–100 ns. The RMSD curve of compound **8**-FGFR3 (black line) is usually stable around 0.28–0.32 nm for 10–100 ns, indicating that compounds **2**, **7**, and **8** can form stable complex systems with FGFR3. The RMSD provides a statistical measure to assess the degree of difference between the predicted structure and the experimentally determined protein structure. It can be seen that these RMSD curves gradually levelled off during the simulation, indicating that the structures of these complexes fluctuated less during the simulation, i.e., the complexes were more stable.

Root-mean-square fluctuation (RMSF) curves are key indicators of the dynamic behaviour of individual amino acid residues in proteins during molecular dynamics simulations [34]. As illustrated in Figure 6B, BRAF exhibits greater residue flexibility at residues 470–485, 490–495, 530–555, 590–660, 530–555, 590–610, and 620–635. As illustrated in Figure 7B, FGFR3 displays greater residue flexibility at residues. The following ranges were identified as exhibiting greater residue flexibility: 470–480, 490–500, 510–520, 540–550, 570–590, and 640–660. The analysis of the RMSF curves enables a more comprehensive understanding of the dynamic behaviour of the receptor protein during molecular dynamics simulations, particularly in relation to residue fluctuations upon binding of small molecules to the protein. These highly fluctuating residues may play a significant role in the function and activity of proteins, providing a valuable reference for subsequent biochemical studies and drug design. 

The radius of gyration (Rg) is a crucial parameter for evaluating the structural compactness and stability of proteins or other biomolecules [35]. Figure 6C demonstrates that the Rg value of BRAF remains stable over 10–100 ns, with the lowest average Rg value ranging from 2.68 to 2.80 nm. The Rg values of compound **2** (blue line) are shown to be stable over 35–100 ns, with the lowest average Rg values ranging from 2.68 to 2.75 nm. The Rg value of compound **7** (red line) remained stable over 0–100 ns, with the lowest average Rg value ranging from 2.67 to 2.75 nm. The Rg value of compound **8** (black line) remained stable over 35–100 ns, with the lowest average Rg value ranging between 2.72 and 2.80 nm. Figure 7C illustrates that the Rg value of FGFR3 remains consistent within the 30–100 ns timeframe, with the lowest average Rg value ranging from 1.98 to 2.08 nm. The Rg value of compound **2** (blue line) remained stable over 70–100 ns, with the lowest average Rg value ranging from 1.97 to 2.07 nm. The Rg value of compound **7** (red line) was shown to be stable over 30–100 ns, with the lowest average Rg value ranging from 1.97 to 2.06 nm. The Rg value of compound **8** (black line) is shown to be stable over 60–100 ns, with the lowest average Rg value ranging from 2.00 to 2.08 nm. This indicates that the protein complex retained a relatively stable spatial conformation throughout the kinetic simulations, demonstrating minimal structural expansion or contraction. This result is crucial for elucidating the stability and interaction mechanisms of the complex. The smooth radius of gyration curves suggest that the complexes were able to maintain their structural integrity during the simulations.

In order to gain a more detailed understanding of the conformational changes of the two proteins during the interaction, we conducted an exhaustive analysis of the 100-nanosecond molecular dynamics simulation data, with a particular focus on the hydrogen bonding formed between the two proteins. As illustrated in Figure 7D. The number of hydrogen bonds exhibited fluctuations throughout the simulation. At the initial stage (0 to 25 ns), the number of hydrogen bonds was relatively low, with an average of between 1 and 4. As the simulation progresses, the number of hydrogen bonds gradually increases, reaching a peak value between 7 and 10 between 20 and 60 ns. This phenomenon indicates that the protein-compound interaction is enhanced during this time period, resulting in the formation of additional hydrogen bonds. This result indicates the existence of a stable yet dynamic hydrogen bonding network between the two proteins. Despite the fluctuations in the number of hydrogen bonds, the overall number remained at a relatively high level, indicating a stronger and more stable interaction between the proteins. This dynamic equilibrium is of great importance in maintaining the overall structure of the protein complex and ensuring its proper function.

#### 2.4.2. Gibbs Free Energy Analysis 

**Figure 8:**
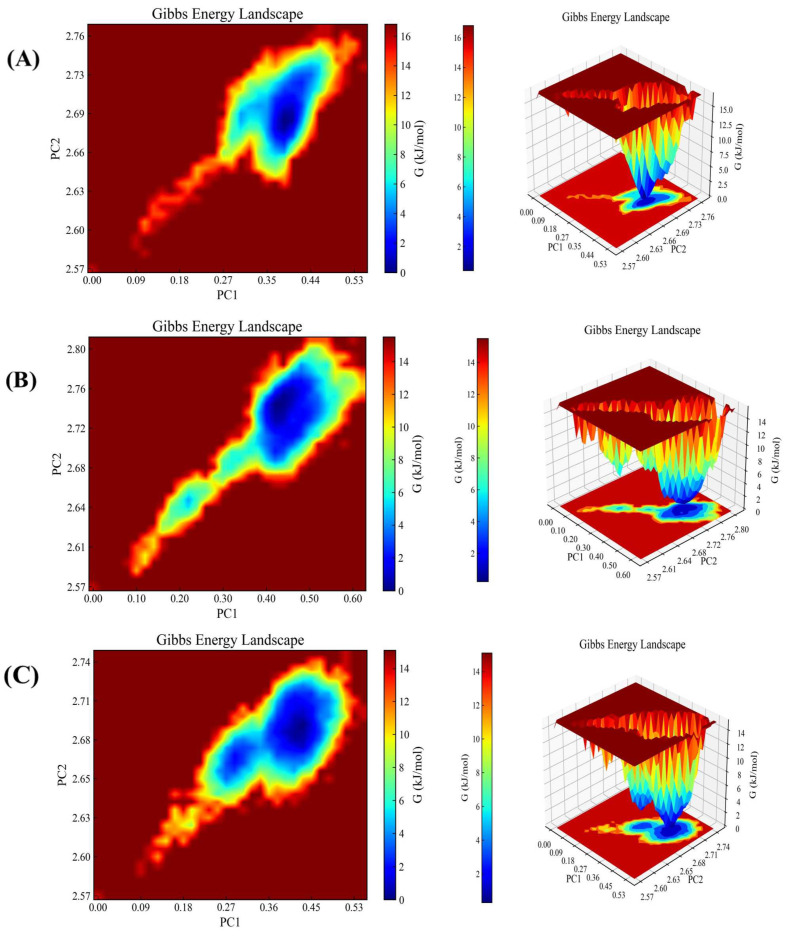
Gibbs free energy analysis. (**A**) compound **2** with BRAF. (**B**) compound **4** with BRAF. (**C**) compound **6** with BRAF.

**Figure 9:**
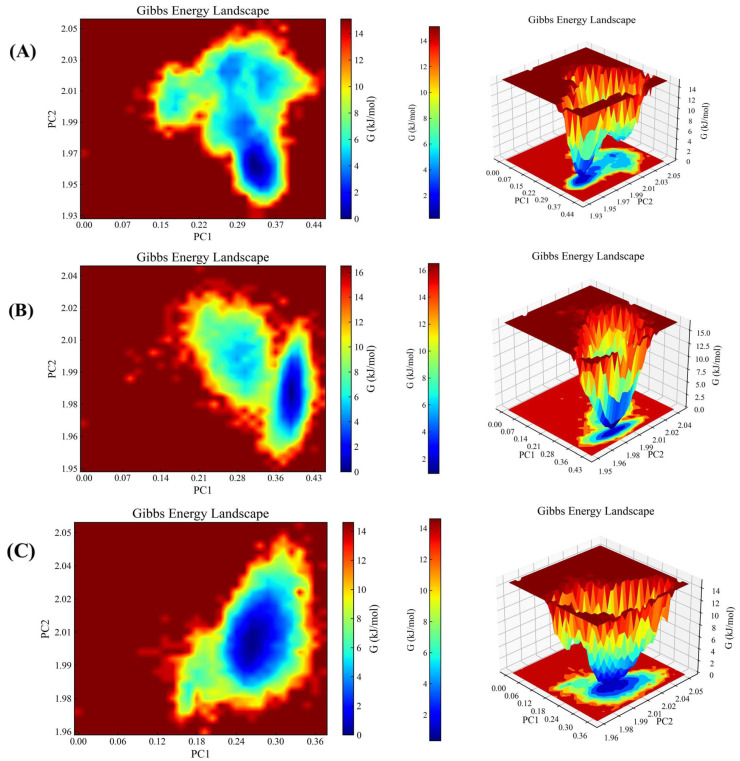
Gibbs free energy analysis. (**A**) is compound **2** with FGFR3. (**B**) compound **7** with FGFR3. (**C**) is compound **8** with FGFR3.

In this study, advanced computational methods and visualisation tools have been employed in order to conduct a comprehensive investigation into the stability and interaction mechanisms of small molecule-protein receptor docking complexes [36,37]. This was based on the RMSD (root-mean-square deviation) and Rg (radius of gyration) values of the complexes. The Gibbs free energy was calculated accurately. As illustrated in Figure 8, compounds **2**, **4**, and **6** exhibit a steady state with the BRAF protein at PC1 0.23–0.50 and PC2 2.64–2.77. The region with a blue hue in the figure prominently labels the steady-state conformations of the complexes, which are clustered at lower energies within the minimum free energy region. As illustrated in Figure 9, compounds **2**, **7**, and **8** exhibit a stable interaction with the FGFR3 protein at PC1 0.22–0.50 and PC2 1.94–2.04. The smoothness and depth of this region reflect the strength of its complex stability. This further corroborates the excellent stability of small-molecule protein receptor complexes, which is crucial for drug design and biomedical research. The complexes are able to maintain their structural and functional integrity under physiological conditions, thus exerting their biological activities more efficiently.

#### 2.4.3. MM/GBSA and Binding Free Energy Analysis

In this study, to further understand the degree of binding stability of small molecules to target protein receptor molecules, the MM/GBSA method was used to calculate the binding free energy and the free energy contributions of the amino acid residues [38]. The binding components in the free energy of binding small molecules to protein receptors are summarised in (Table 4 and Table 5). The free energies of binding of Compound **2**-FGFR3, Compound **7**-FGFR3, and Compound **8**-FGFR3 were −44.43 ± 6.00, −37.23 ± 1.93, and −45.87 ± 3.55 kcal/mol, respectively, as shown in Table 4. The binding free energies of Compound **2**-BRAF, Compound **4**-BRAF, and Compound **6**-BRAF were −44.19 ± 2.33 kcal/mol, −51.92 ± 4.19 kcal/mol, and −50.24 ± 2.09 kcal/mol, respectively, as shown in Table 5.

## 3. Discussion

In the present study, 12 sucrose esters were isolated and purified from *Atractylodes japonica*, including five new compounds and seven known compounds. This study greatly enhanced the presence of this class of components in natural products. In addition, the results of the cytotoxic activity demonstrated that these compounds exhibited activity against colon cancer and lung cancer cells. The superior cell toxicity and ability to bind to BRAF and FGFR3 of compounds **1**–**7** in comparison to compounds **8**–**12** suggest that the activity of sucrose esters with three isovaleryl substitutions is superior to that of sucrose esters with five isovaleryl substitutions. This provided a theoretical basis for the subsequent structural modification of these compounds. Additionally, the results of molecular dynamics simulations indicated that the binding energies and thermodynamic stability between the small molecules and protein receptors were enhanced. Moreover, the laboratory synthesis of this class of compounds is relatively straightforward, rendering it highly valuable for intensive research and exploitation.

## 4. Materials and Methods

### 4.1. Plant Materials

The rhizomes of *A. japonica* were collected in October 2021 from the Daxinganling region in Heilongjiang Province, China. Professor Wang Zhenyue authenticated these rhizomes as *Atractylodes japonica*. A specimen has been archived at the School of Pharmacy, Heilongjiang University of Chinese Medicine, Harbin, China, with the reference number 20211007.

### 4.2. General Experimental Procedures

Mass spectrometry analyses were conducted using a 5600^+^ Q-TOF high-resolution mass spectrometer (AB SCIEX, Framingham, MA, USA). Proton and carbon NMR spectra were recorded on a Bruker (Billerica, MA, USA) DPX-300 Fourier transform NMR instrument with operating frequencies of400, 600 and 150 MHz, respectively. Chemical shifts (δ ppm) were referenced to tetramethylsilane. Chromatographic analyses utilised an LC-20AD preparative liquid chromatography (Shimadzu Corporation, Kyoto, Japan) equipped with a semi-preparative Waters (Milford, MA, USA) Sun-Fire Prep C18 column (10 mm × 250 mm, 10 µm). Optical rotations were measured using a Jasco P-2000 digital polarimeter (JASCO Corporation, Kyoto, Japan). Column chromatography was performed with silica gel (200–300 mesh; Qingdao Marine Chemical, Inc., Qingdao, China) and ODS (50 μm, YMC Company Ltd., Kyoto, Japan). NMR spectra were processed with MestReNova software (version 14.0). HCT 116 (CL-0096) and A549 [A-549] (CL-0016) cells were kindly provided by Wuhan Pricella Biotechnology Co., Ltd. (Wuhan, China). The DMEM medium was purchased from Beyotime Ltd. (Shanghai, China).

### 4.3. Extraction, Isolation, and Structure Identification

The rhizome of *A. japonica* was subjected to heating reflux extraction with 75% ethanol, which was then concentrated into an infusion. Sequential extraction with organic solvents, after dispersion in water, yielded PE (AJ1, 140.3 g), DCM (AJ2, 183.7 g), EtOAc (AJ3, 95.2 g), n-butanol (AJ4, 65.5 g), and H_2_O (AJ5, 922.3 g) layers post-in vacuo solvent removal. Silica gel column chromatography of the n-butanol fraction (AJ4, 65.5 g) using gradients of CH_2_Cl_2_-CH_3_OH led to ten subfractions, AJ4A–AJ4J. Subfractions AJ4D (6.0 g) and AJ4G (4.0 g) were further fractionated using ODS column chromatography with a CH_3_OH: H_2_O gradient, resulting in ten smaller fractions each: AJ4D.1–10 and AJ4G.1–10. HPLC of AJ4D.4 using a CH_3_OH-H_2_O (43:57) mobile phase at 3 mL/min yielded compounds **1** (12.1 mg), **2** (8.4 mg), and **5** (13.4 mg). Similarly, AJ4D.6 yielded compounds **3** (8.6 mg), **6** (8.1 mg), **7** (10.5 mg), and **8** (10.9 mg) under CH_3_OH-H_2_O (48:52) conditions. AJ4G.4 processed with CH_3_OH-H_2_O (40:60) yielded compounds **4** (7.2 mg), **9** (9.6 mg), **10** (11.5 mg), and **11** (8.3 mg). In the structural identification of the compounds, the molecular weight of each compound was determined by HR-EI-MS, the structure of the known compounds was determined by comparing 1D-NMR data with the literature, and DEPT spectra were used to differentiate between primary, secondary, tertiary, and quaternary carbons in the carbon diagrams in the resolution of the new compounds; the directly connected carbon-hydrogen relationships were determined by HSQC spectra; and the relative configurations were identified by HHCOSY and HMBC spectra. The relative configurations were determined by NOESY spectra.

### 4.4. Cytotoxicity Assays

The human colorectal carcinoma (HCT-116) and human non-small cell lung carcinoma (A549) cells were cultured in high-glucose Dulbecco’s modified Eagle’s medium (DMEM) supplemented with 10% foetal bovine serum (FBS) and 1% penicillin-streptomycin. In brief, the two types of cells were seeded in flat-bottomed 96-well microplates at a density of 4000 cells per well, with a total volume of 0.2 mL per well. Following a 24-h incubation period, the cells were exposed to varying concentrations of the compounds (1–12) for a further 48 h. The concentration range of the compounds was between 0 and 100 μM. After 24 h of incubation, the existing medium was replaced with 100 μL of fresh medium containing 0.5 mg/mL of CCK-8 reagent. Subsequently, the cells were incubated for a further two hours under the same conditions. Finally, the absorbance was measured at 490 nm in order to calculate the IC_50_ value, as previously reported in the literature [39]. The IC_50_ value was deemed to be greater than 50 μM, indicating that the compound was inactive or ineffective. Doxorubicin was used as a positive control.

### 4.5. Molecular Docking Analysis

The crystal structures of FGFR3 (PDB ID: 6LVM) and BRAF (PDB ID: 8C7X) were obtained from UniProt (https://www.uniprot.org, accessed on 2 April 2024). The receptor was prepared using PyMOL 2.5.5 and deposited in .pdb format. The 3D structures of the ligands were energy optimised using ChemDraw 3D 14.0 and deposited in mol2 format. The ligands and receptors for molecular docking analysis were prepared using AutoDock Vina 1.2.2 [33]. The 6LVM grid box parameters (X centre = 21,435, Y centre = 85,695, Z centre = 17,872; x dimension = 20, y dimension = 20, z dimension = 20). The parameters of the 8C7X grid box (X centre = 4.270, Y centre = −4.177, Z centre = 11.542; x dimension = 40, y dimension = 40, z dimension = 40) were set to cover the binding pocket in the receptor. The results of the docking calculations were analysed using PyMOL 2.5.5 [39,40,41]. 2D visualisation analysis was performed using ligplus 2.2.8 software [42].

### 4.6. Molecular Dynamics Simulation Analysis

Molecular dynamics simulations were conducted to evaluate the binding stability, conformation, and interaction pattern of compounds **2**, **7**, **8** with the receptor FGFR3 (PDBID: 6LVM) and compounds **2**, **4**, and **6** with the receptor BRAF (PDBID: 8C7X), respectively. The software employed was Gromacs 2022.3 [43,44], with the simulation conditions maintained at a static temperature of 300 K. The force field was Amber99sb-ildn, the solvent was a water molecule (Tip3p water model), and the equilibrium duration was 100 ps. Finally, molecular dynamics simulations were carried out for 100 ns. Finally, we analysed each set of molecular dynamics simulation trajectories for the root mean square deviation (RMSD), root mean square fluctuation (RMSF), radius of gyration (Rg), number of hydrogen bonds, and Gibbs free energy diagrams. These analyses were conducted in accordance with the methodologies outlined in references [45,46,47,48].

## 5. Conclusions

In the present study, 12 sucrose esters were isolated and purified from *Atractylodes japonica*, including five new compounds and seven known compounds. The results of cytotoxicity assays show that compounds **2**, **4**, and **6** were moderately inhibitory to HCT-116 cells, with IC_50_ values of 7.49 ± 0.48, 9.03 ± 0.21, and 13.49 ± 1.45 μM, respectively. Compounds **1** and **6** were moderately inhibitory to A549, with IC_50_ values of 8.36 ± 0.77 and 7.10 ± 0.52 μM, respectively. Furthermore, the findings of molecular docking and molecular dynamics simulations indicate that these components may be associated with the FGFR3 and BRAF proteins.

## Figures and Tables

**Table 1:** ^1^H-NMR spectroscopic data of compounds **1**–**5** (*δ* in ppm, *J* in Hz).

No	1 ^a^	2 ^a^	3 ^b^	4 ^b^	5 ^b^
1	5.53 (d, 3.5)	5.54 (d, 3.7)	5.50 (d, 3.8)	5.50 (d, 3.8)	5.53 (d, 3.5)
2	3.73 m	4.77 (dd, 10.1, 3.7)	4.63 (dd, 10.1, 3.8)	4.62 (dd, 10.2, 3.8)	3.73 m
3	3.81 m	3.99 m	3.73 m	3.77 m	5.32 (t, 9.8)
4	4.86 (t, 9.6)	3.59 m	3.38 (t, 9.1)	3.44 m	5.08 (t, 9.8)
5	4.00 m	3.95 m	4.01 m	3.86 m	4.16 m
6	3.75 m	3.88 m	4. 45 (dd, 12.0, 2.0)	3.84 m	3.75 m
	3.58 m	3.82 m	4.16 (dd, 12.0, 5.3)	3.79 m	3.58 m
1′	3.65 m	3.88 m	3.35 (d, 11.8)	3.50 m	3.79 m
	3.70 m	3.72 m	3.46 (d, 11.8)	3.37 m	3.77 m
3′	5.37 (d, 5.9)	5.48 (d, 7.4)	5.43 (d, 8.6)	5.45 (d, 8.6)	5.64 (d, 7.2)
4′	5.43 (t, 5.9)	5.45 (t, 7.1)	4.23 (t, 8.6)	4.32 (t, 8.5)	5.48 (t, 7.3)
5′	4.08 m	4.08 m	3.88 m	4.08 m	4.13 m
6′	3.78 m	3.63 m	3.77 m	4.35m	3.56 m
	3.67 m				3.65 (d, 12.4)
2″	2.21–2.31 m	2.15–2.42 m	2.18–2.36 m	2.19–2.32 m	2.30–2.22 m
3″	2.06–2.16 m	1.98–2.13 m	2.14–2.16 m	2.04–2.18 m	2.09–2.02 m
4″/5″	0.89–1.02 m	0.99–0.90 m	0.96–1.02 m	0.95–1.01 m	1.02–0.96 m

Assignments aided by the DEPT (Distortionless Enhancement by Polarisation Transfer), ^1^H-^1^H COSY (^1^H-^1^H Correlated Spectroscopy), HSQC (H-detected heteronuclear Single-quantum Correlation), HMBC (H-detected heteronuclear Multiple-Bond Correlation), and NOESY (Nuclear Overhauser Enhancement Spectroscopy) experiments; The unit of chemical shift (*δ*) for all above parameters is ppm; ^a^ measured in CDCl_3_, ^b^ measured in CD_3_OD. Compounds **1**, **2**, and **5** measured in 600 MHz, compounds **3** and **4** measured in 400 MHz.

**Table 2:** ^13^C-NMR (150 MHz) spectroscopic data of compounds **1**–**5** (*δ* in ppm).

No.	1 ^a^	2 ^a^	3 ^b^	4 ^b^	5 ^b^
1	92.0	90.7	90.6	90.6	93.1
2	72.1	72.3	73.8	74.1	70.9
3	71.9	72.9	72.2	72.3	74.0
4	70.3	70.4	71.7	71.6	69.7
5	72.2	71.5	71.9	74.1	72.2
6	61.2	61.4	64.4	62.4	61.3
1′	61.1	62.0	64.4	64.3	63.2
2′	104.5	104.6	104.9	105.1	105.4
3′	77.7	76.1	78.1	77.8	77.1
4′	74.2	73.5	73.6	74.2	75.8
5′	82.0	81.4	84.3	80.9	82.5
6′	64.9	63.8	63.8	65.9	64.6
1″	173.7	173.4	174.7	174.7	174.0
	172.6	172.6	174.2	174.1	173.8
	172.3	172.5	174.1	174.0	173.4
					173.3
2″	43.5	43.2	44.1 × 3	44.1	44.2 × 2
	43.1 × 2	43.1		44.0 × 2	43.9 × 2
		42.8			
3″	25.9	25.7 × 2	27.0	27.0	26.8
	25.7 × 2	25.5	26.9	26.8	26.6 × 2
			26.5	26.7	26.5
4″/5″	22.6 × 2	22.4 × 3	22.9	22.9	22.9 × 2
	22.5	22.3 × 3	22.8 × 3	22.8 × 3	22.8 × 2
	22.4 × 3		22.7 × 2	22.7 × 2	22.7 × 2
					22.6 × 2

Assignments aided by the DEPT (Distrotionless Enhancement by Polarisation Transfer), ^1^H-^1^H COSY (^1^H-^1^H Correlated Spectroscopy), HSQC (H-detected heteronuclear Single-quantum Correlation), HMBC (H-detected heteronuclear Multiple-Bond Correlation), and NOESY (Nuclear Overhauser Enhancement Spectroscopy) experiments; The unit of chemical shift (*δ*) for all above parameters is ppm; ^a^ measured in CDCl_3_, ^b^ measured in CD_3_OD.

**Table 3:** Cytotoxic activities of compounds **1**–**12** (mean ± SD, *n* = 3).

IC_50_ (μM)					
No.	Hct116	A549	No.	Hct116	A549
**1**	18.77 ± 1.56	8.36 ± 0.77	**8**	20.10 ± 0.26	22.08 ± 2.19
**2**	7.49 ± 0.48	15.60 ± 0.53	**9**	24.75 ± 0.24	18.25 ± 1.98
**3**	21.75 ± 0.24	12.26 ± 1.17	**10**	>50	40.50 ± 0.76
**4**	9.03 ± 0.21	21.95 ± 0.33	**11**	>50	>50
**5**	22.06 ± 0.68	30.58 ± 2.22	**12**	>50	32.60 ± 0.69
**6**	13.49 ± 1.45	7.10 ± 0.52	Doxorubicin	2.14 ± 1.08	1.78 ± 0.56
**7**	21.65 ± 0.40	18.55 ± 1.56			

**Table 4:** Combined free energy calculations of compounds **2**, **7**, and **8** with FGFR3 (PDB ID:6LVM).

	Compound 2-FGFR3	Compound 7-FGFR3	Compound 8-FGFR3
VDWAALS	−54.70 ± 0.04	−46.64 ± 1.04	−59.22 ± 1.40
ΔEEl	−40.18 ± 5.61	−25.16 ± 1.60	−30.25 ± 2.92
ΔEGB	58.51 ± 2.13	41.21 ± 0.29	51.72 ± 1.43
ΔEsurf	−8.06 ± 0.09	−6.64 ± 0.06	−8.12 ± 0.14
ΔGgas	−94.88 ± 5.61	−71.80 ± 1.91	−89.47 ± 3.24
ΔGsolvation	50.45 ± 2.13	34.57 ± 0.30	43.60 ± 1.44
ΔTotal	−44.43 ± 6.00	−37.23 ± 1.93	−45.87 ± 3.55

**Table 5:** Combined free energy calculations of compounds **2**, **4**, and **6** with BRAF (PDB ID: 8C7X).

	Compound 2-BRAF	Compound 4-BRAF	Compound 6-BRAF
VDWAALS	−59.33 ± 0.60	−61.31 ± 1.73	−57.46 ± 0.31
ΔEEl	−29.46 ± 2.19	−37.77 ± 3.24	−36.74 ± 2.05
ΔEGB	53.19 ± 0.49	55.22 ± 2.02	51.97 ± 0.21
ΔEsurf	−8.59 ± 0.01	−8.07 ± 0.00	−8.01 ± 0.07
ΔGgas	−88.79 ± 2.27	−99.08 ± 3.68	−94.19 ± 2.08
ΔGsolvation	44.60 ± 0.49	47.15 ± 2.02	43.96 ± 0.23
ΔTotal	−44.19 ± 2.33	−51.92 ± 4.19	−50.24 ± 2.09

## Data Availability

The data presented in this study are available in this article.

## Supplementary Materials

The following supporting information can be downloaded at: https://www.mdpi.com/article/10.3390/molecules29133069/s1, Figures S1–S45, Table S1 and S2: 1D, 2D NMR, HRESIMS, IR, and UV spectra of compounds **1**–**5**, ^1^H-NMR, and ^13^C-NMR data for compounds **6**–**12**.

## References

[1] Sun J., Zhao J., Jiang F., Wang L., Xiao Q., Han F., Chen J., Yuan S., Wei J., Larsson S.C. (2023). Identification of novel protein biomarkers and drug targets for colorectal cancer by integrating human plasma proteome with genome. Genome Med..

[2] Zhu M., Sun Y., Bai H., Wang Y., Yang B., Wang Q., Kuang H. (2023). Effects of saponins from Chinese herbal medicines on signal transduction pathways in cancer: A review. Front. Pharmacol..

[3] Chrencik J.E., Staker B.L., Burgin A.B., Pourquier P., Pommier Y., Stewart L., Redinbo M.R. (2004). Mechanisms of camptothecin resistance by human topoisomerase I mutations. J. Mol. Biol..

[4] Zhu Y., Wang A., Zhang S., Kim J., Xia J., Zhang F., Wang D., Wang Q., Wang J. (2023). Paclitaxel-loaded ginsenoside Rg3 liposomes for drug-resistant cancer therapy by dual targeting of the tumor microenvironment and cancer cells. J. Adv. Res..

[5] Jiang X.Y., Shi L.P., Zhu J.L., Bai R.R., Xie T. (2024). Elemene Antitumor Drugs Development Based on “Molecular Compatibility Theory” and Clinical Application: A Retrospective and Prospective Outlook. Chin. J. Integr. Med..

[6] Zhao Q.L., Wang M.J., Zhao M., Zheng B.J. (2018). Research progress on *Atractylodes japonica*. Chin. Tradit. Herb. Drugs.

[7] Choi E.M., Kim G.H., Lee Y.S. (2009). *Atractylodes japonica* root extract protects osteoblastic MC3T3-E1 cells against hydrogen peroxide-induced inhibition of osteoblastic differentiation. Phytother. Res..

[8] Yamamoto K., Yamashita K., Hitomi N., Suzuki A., Oneda K. (1993). Studies on the constituents of *Atractylodes rhizome*, constituents in the rhizome of *Atractylodes japonica* and TLC analysis of Jutsu. Jpn. J. Pharmacogn..

[9] Daudé D., Remaud-Siméon M., André I. (2012). Sucrose analogs: An attractive (bio)source for glycodiversification. Nat. Prod. Rep..

[10] Teng Y., Stewart S.G., Hai Y.W., Li X., Banwell M.G., Lan P. (2021). Sucrose fatty acid esters: Synthesis, emulsifying capacities, biological activities and structure-property profiles. Crit. Rev. Food. Sci. Nutr..

[11] Murakami N., Iwata E., Tamura S., Akiyama S., Kobayashi M. (2000). New multidrug resistance modulators from *Atractylodis lanceae* rhizoma. Bioorg. Med. Chem. Lett..

[12] Tanaka K., Ina A. (2009). Structure elucidation of acylsucrose derivatives from *Atractylodes lanceae* rhizome and *Atractylodes rhizome*. Nat. Prod. Commun..

[13] Fang X., Zhuo Z.G., Xu X.K., Ye J., Li H.L., Shen Y.H., Zhang W.D. (2017). Cytotoxic isovaleryl sucrose esters from Ainsliaea yunnanensis: Reduction of mitochondrial membrane potential and increase of reactive oxygen species levels in A549 cells. RSC Adv..

[14] Tchinda A.T., Tane P., Ayafor J.F., Connolly J.D. (2003). Stigmastane derivatives and isovaleryl sucrose esters from *Vernonia guineensis* (Asteraceae). Phytochemistry.

[15] Wu Q., Cho J.G., Lee D.S., Lee D.Y., Song N.Y., Kim Y.C., Lee K.T., Chung H.G., Choi M.S., Jeong T.S. (2013). Carbohydrate derivatives from the roots of *Brassica rapa* ssp. campestris and their effects on ROS production and glutamate-induced cell death in HT-22 cells. Carbohydr. Res..

[16] Kim C.T., Jung M.H., Kim H.S., Kim H.J., Kang S.H. (2000). Inhibitors of melanogenesis from *Euphorbieae lathyridis* Semen. Korean J. Pharmacogn..

[17] Ullah R., Yin Q., Snell A.H., Wan L. (2022). RAF-MEK-ERK pathway in cancer evolution and treatment. Semin. Cancer Biol..

[18] Liu G., Chen T., Ding Z., Wang Y., Wei Y., Wei X. (2021). Inhibition of FGF-FGFR and VEGF-VEGFR signalling in cancer treatment. Cell Prolif..

[19] Molina-Cerrillo J., San Román M., Pozas J., Alonso-Gordoa T., Pozas M., Conde E., Rosas M., Grande E., García-Bermejo M.L., Carrato A. (2020). BRAF Mutated Colorectal Cancer: New Treatment Approaches. Cancers.

[20] Johansson C.H., Brage S.E. (2014). BRAF inhibitors in cancer therapy. Pharmacol. Ther..

[21] Zhang Y., Wang D.C., Shi L., Zhu B., Min Z., Jin J. (2017). Genome analyses identify the genetic modification of lung cancer subtypes. Semin. Cancer Biol..

[22] Chen Y., Zhang M., Wu A., Yao X., Wang Q. (2022). Structure-Based Discovery and Biological Assays of a Novel PRMT5 Inhibitor for Non-Small Cell Lung Cancer. Molecules.

[23] Saif M.W., Elfiky A., Salem R.R. (2007). Gastrointestinal perforation due to bevacizumab in colorectal cancer. Ann. Surg. Oncol..

[24] Prahallad A., Sun C., Huang S., Di Nicolantonio F., Salazar R., Zecchin D., Beijersbergen R.L., Bardelli A. (2012). Unresponsiveness of colon cancer to BRAF(V600E) inhibition through feedback activation of EGFR. Nature.

[25] Desai A., Adjei A.A. (2016). FGFR Signaling as a Target for Lung Cancer Therapy. J. Thorac. Oncol..

[26] Subbiah V., Iannotti N.O., Gutierrez M., Smith D.C., Féliz L., Lihou C.F., Tian C., Silverman I.M., Ji T., Saleh M. (2022). FIGHT-101, a first-in-human study of potent and selective FGFR 1–3 inhibitor pemigatinib in pan-cancer patients with FGF/FGFR alterations and advanced malignancies. Ann. Oncol..

[27] Helsten T., Elkin S., Arthur E., Tomson B.N., Carter J., Kurzrock R. (2016). The FGFR Landscape in Cancer: Analysis of 4853 Tumors by Next-Generation Sequencing. Clin. Cancer Res..

[28] Bock K., Pedersen C., Pedersen H. (1984). Carbon-13 nuclear magnetic resonance data for oligosaccharides. Adv. Carbohydr. Chem. Biochem..

[29] Tabopda T.K., Mitaine-Offer A.C., Paululat T., Delemasure S., Dutartre P., Ngadjui B.T., Lacaille-Dubois M.A. (2016). Steroidal saponins from *Chlorophytum deistelianum*. Phytochemistry.

[30] Nana H., Penglonh W., Hongshan C. (2022). Basic research on antioxidant substances of gentiana macrophylla based on composition analysis-activity screening-network pharmacology. Chin. J. Tradit. Chin. Med..

[31] Arora R., Linders J.T.M., Aci-Sèche S., Verheyen T., Van Heerde E., Brehmer D., Chaikuad A., Knapp S., Bonnet P. (2023). Design, synthesis and characterisation of a novel type II B-RAF paradox breaker inhibitor. Eur. J. Med. Chem..

[32] Kuriwaki I., Kameda M., Hisamichi H., Kikuchi S., Iikubo K., Kawamoto Y., Moritomo H., Kondoh Y., Amano Y., Tateishi Y. (2020). Structure-based drug design of 1,3,5-triazine and pyrimidine derivatives as novel FGFR3 inhibitors with high selectivity over VEGFR2. Bioorg. Med. Chem..

[33] Jiang X.Z., Ventikos Y. (2022). Molecular dynamics simulation: A new way to understand the functionality of the endothelial glycocalyx. Curr. Opin. Struct. Biol..

[34] Zhu J., Li K., Xu L., Cai Y., Chen Y., Zhao X., Li H., Huang G., Jin J. (2021). Discovery of novel selective PI3Kγ inhibitors through combining machine learning-based virtual screening with multiple protein structures and bio-evaluation. J. Adv. Res..

[35] Dorado G., Gálvez S., Rosales T.E., Vásquez V.F., Hernández P. (2021). Analyzing Modern Biomolecules: The Revolution of Nucleic-Acid Sequencing—Review. Biomolecules.

[36] Salike S., Bhatt N. (2020). Thermodynamically consistent estimation of Gibbs free energy from data: Data reconciliation approach. Bioinformatics.

[37] Jia Z.J., Lan X.W., Lu K., Meng X., Jing W.J., Jia S.R., Zhao K., Dai Y.J. (2023). Synthesis, molecular docking, and binding Gibbs free energy calculation of β-nitrostyrene derivatives: Potential inhibitors of SARS-CoV-2 3CL protease. J. Mol. Struct..

[38] Dasmahapatra U., Kumar C.K., Das S., Subramanian P.T., Murali P., Isaac A.E., Ramanathan K., Mm B., Chanda K. (2022). In-silico molecular modelling, MM/GBSA binding free energy and molecular dynamics simulation study of novel pyrido fused imidazo[4,5-c] quinolines as potential anti-tumor agents. Front. Chem..

[39] Liu L.F., Sun H.H., Tan J.B., Huang Q., Cheng F., Xu K.P., Zou Z.X., Tan G.S. (2021). New cytotoxic biflavones from *Selaginella doederleinii*. Nat. Prod. Res..

[40] Ashiru M.A., Ogunyemi S.O., Temionu O.R., Ajibare A.C., Cicero-Mfon N.C., Ihekuna O.A., Jagun M.O., Abdulmumin L., Adisa Q.K., Asibor Y.E. (2023). Identification of EGFR inhibitors as potential agents for cancer therapy: Pharmacophore-based modeling, molecular docking, and molecular dynamics investigations. J. Mol. Model..

[41] Huang L., Wang Z., Wang F., Wang S., Wang D., Gao M., Li H., Song M., Zhang X. (2024). Triterpenoids from the Leaves of Diospyros digyna and Their PTP1B Inhibitory Activity. Molecules.

[42] Wang N., Li Q. (2023). Simultaneous Extraction and Analysis of Seven Major Saikosaponins from Bupleuri Radix and the Exploration of Antioxidant Activity and Its Mechanism. Molecules.

[43] Abraham M.J., Murtola T., Schulz R. (2015). GROMACS: High performance molecular simulations through multi-level parallelism from laptops to supercomputers. SoftwareX.

[44] Van Der Spoel D., Lindahl E., Hess B. (2005). GROMACS: Fast, flexible, and free. J. Comput. Chem..

[45] Lobato-Tapia C.A., Moreno-Hernández Y., Olivo-Vidal Z.E. (2023). In Silico Studies of Four Compounds of *Cecropia obtusifolia* against Malaria Parasite. Molecules.

[46] Filipe H.A.L., Loura L.M.S. (2022). Molecular Dynamics Simulations: Advances and Applications. Molecules.

[47] Hu X., Zeng Z., Zhang J., Wu D., Li H., Geng F. (2023). Molecular dynamics simulation of the interaction of food proteins with small molecules. Food Chem..

[48] Ouyang J., Hu N., Wang H. (2024). Isolation, Purification and Tyrosinase Inhibitory Activity of Anthocyanins and Their Novel Degradation Compounds from *Solanum tuberosum* L.. Molecules.

