# Supplementary material for: Isovaleryl Sucrose Esters from Atractylodes japonica and Their Cytotoxic Activity
Source: Molecules. 2024 Jun 27;29(13):3069. doi: 10.3390/molecules29133069 (PMC11243297; doi:10.3390/molecules29133069)
Supplement: Supplementary file 1 [file molecules-29-03069-s001.zip › molecules-3042964-supplementary-figures updated.pdf]

Supplementary Materials

Isovaleryl sucrose esters from *Atractylodes japonica* and their cytotoxic activity

Yi-meng Wang, Zhi-bin Wang, Yan-ping Sun, Ming-tao Zhu, Yong Jiang, Hao-dong Bai, Bing-you Yang, and Hai-xue Kuang*

Key Laboratory of Basic and Application Research of Beiyao, Ministry of Education, Heilongjiang University of Chinese Medicine, Harbin 150040, China.

*Corresponding Author: hxkuang@hljucm.edu.cn

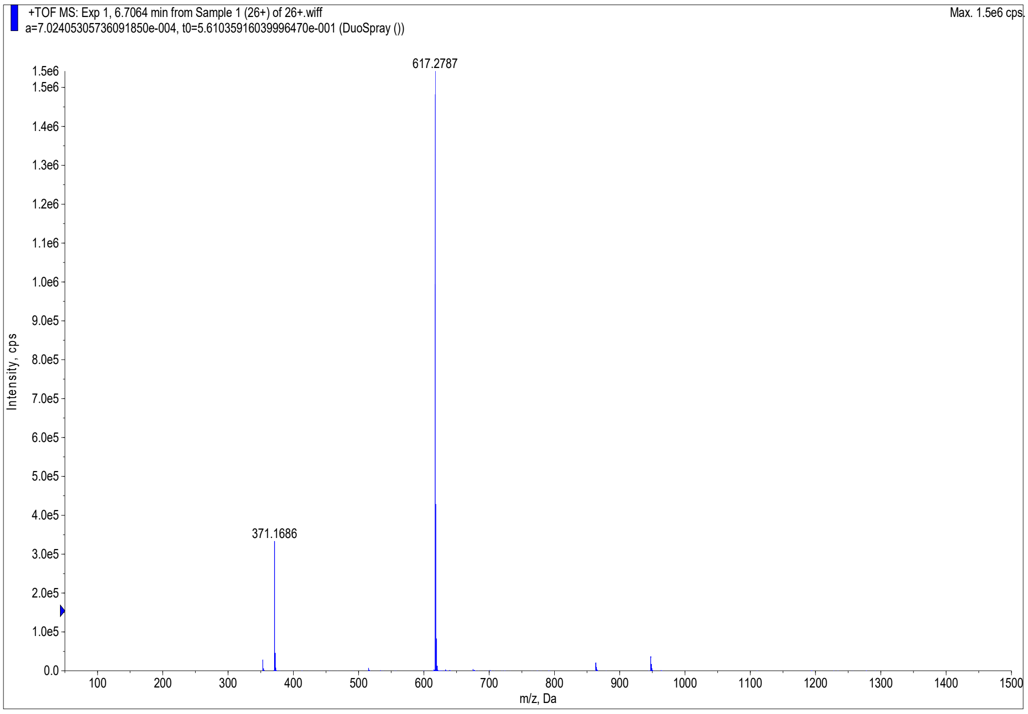

Figure S1: HR-ESI-MS spectrum of compound **1**

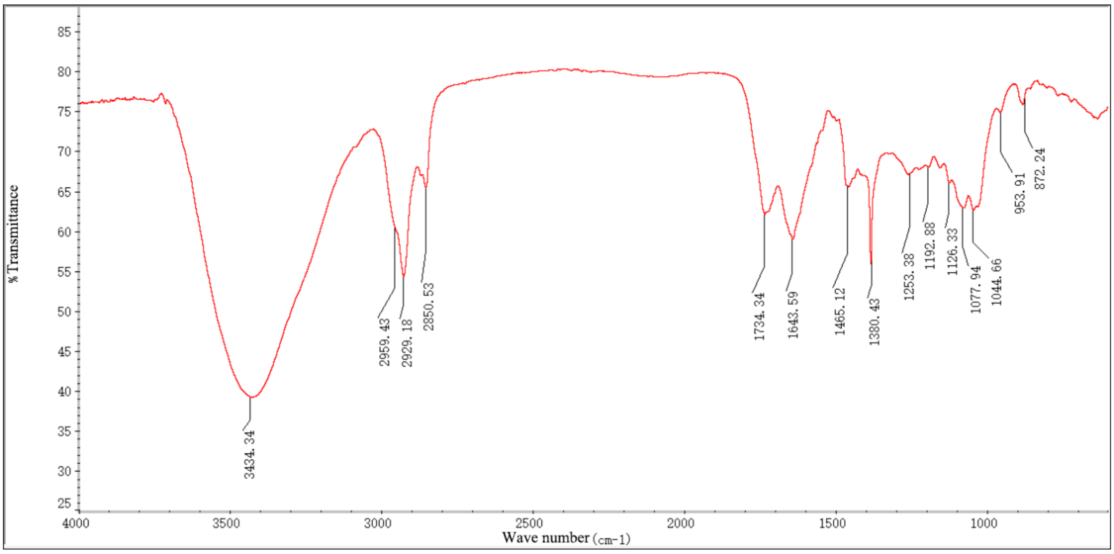

Figure S2: IR spectrum of compound **1**

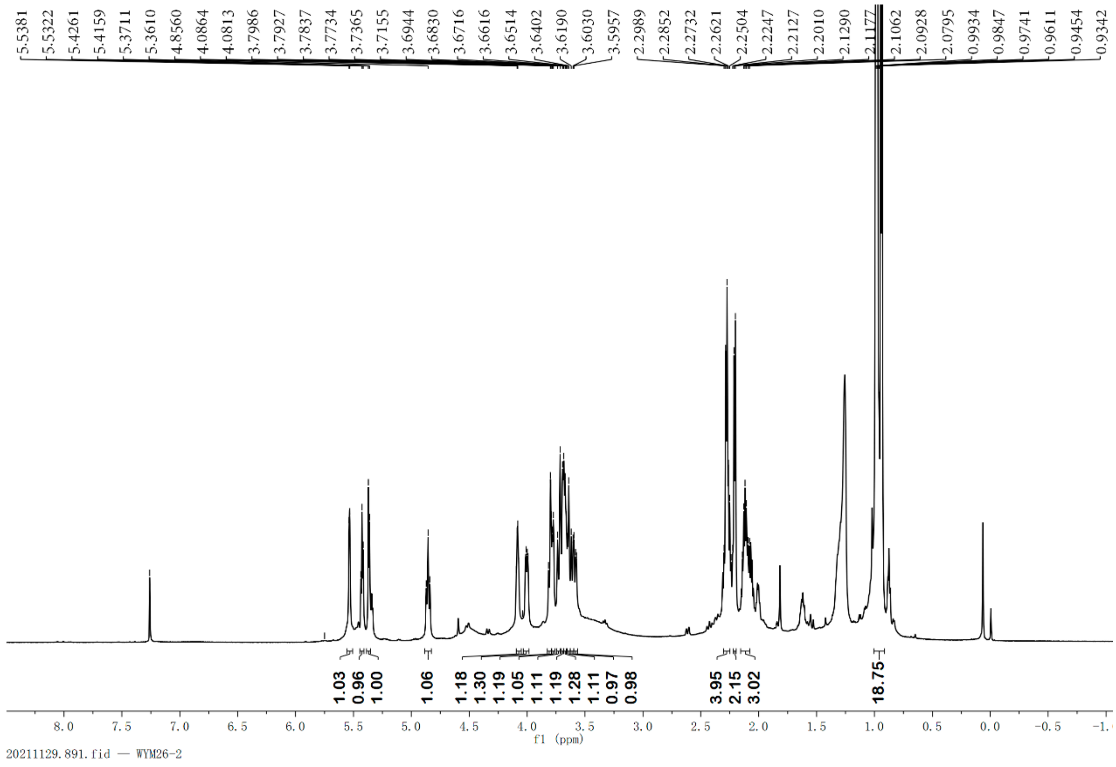

Figure S3: ^{1}H-NMR spectrum of compound **1** (600 MHz, $CDCl_3$)

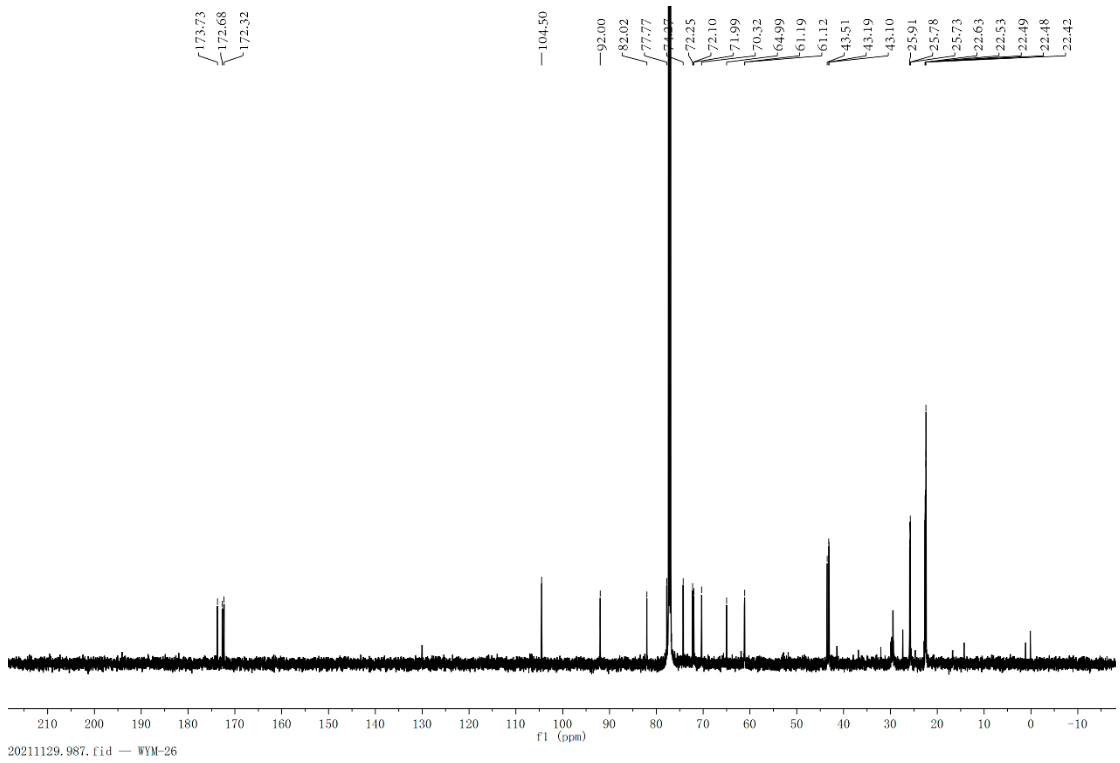

Figure S4: ^{13}C-NMR spectrum of compound **1** (150 MHz, $CDCl_3$)

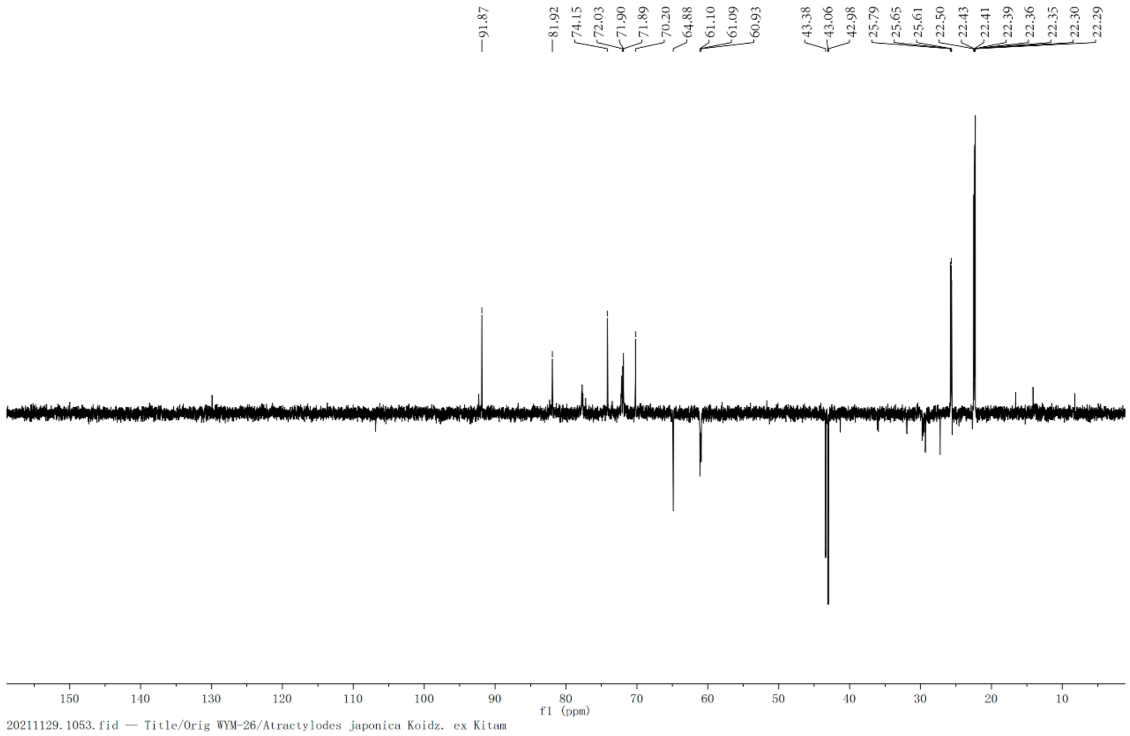

Figure S5: DEPT 135° NMR spectrum of compound **1**

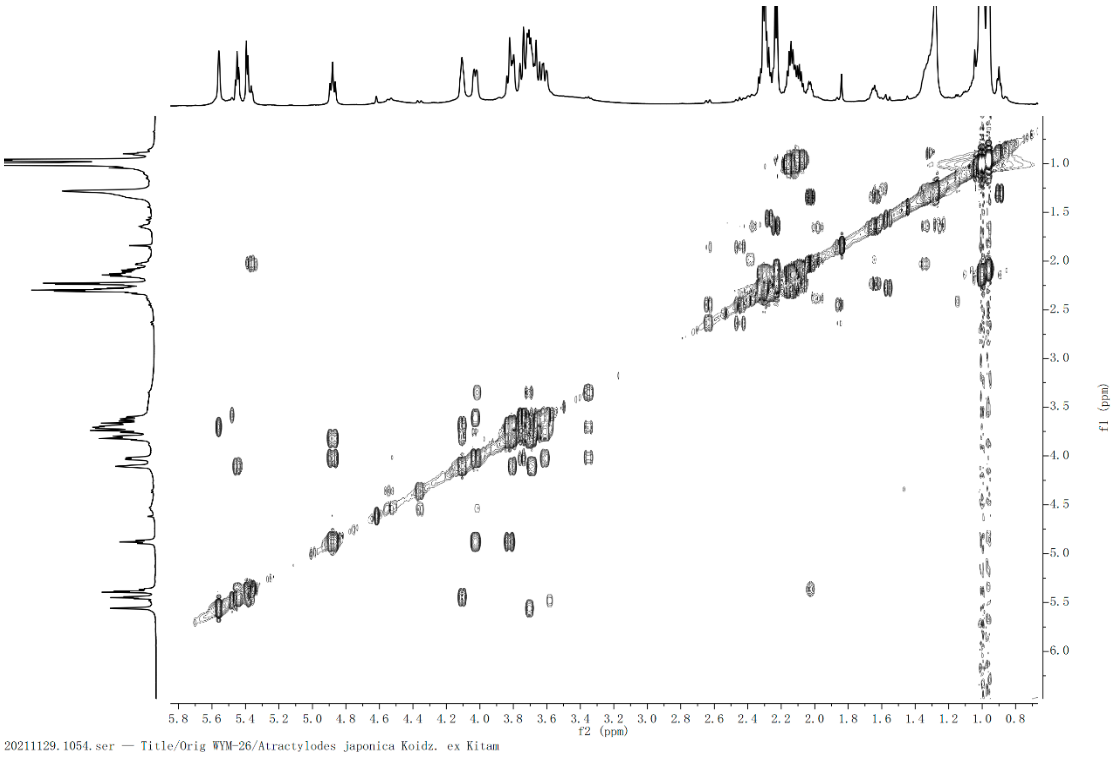

Figure S6: ^{1}H-^{1}H COSY NMR spectrum of compound **1**

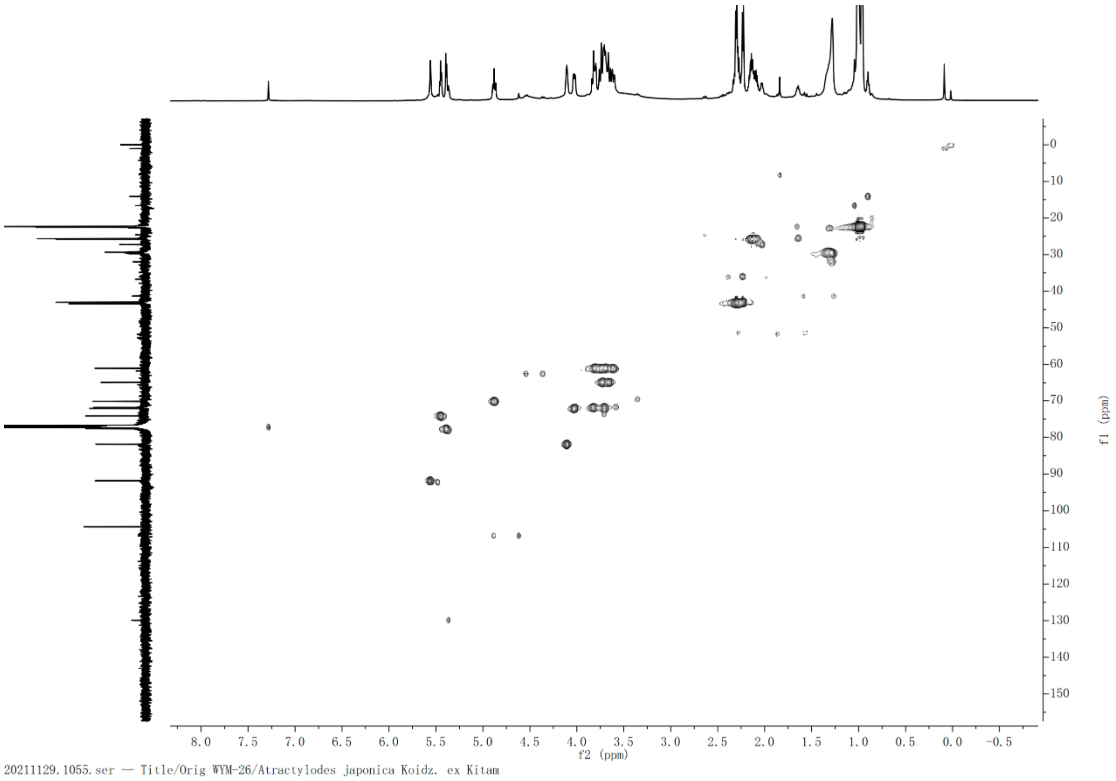

Figure S7: HSQC NMR spectrum of compound **1**

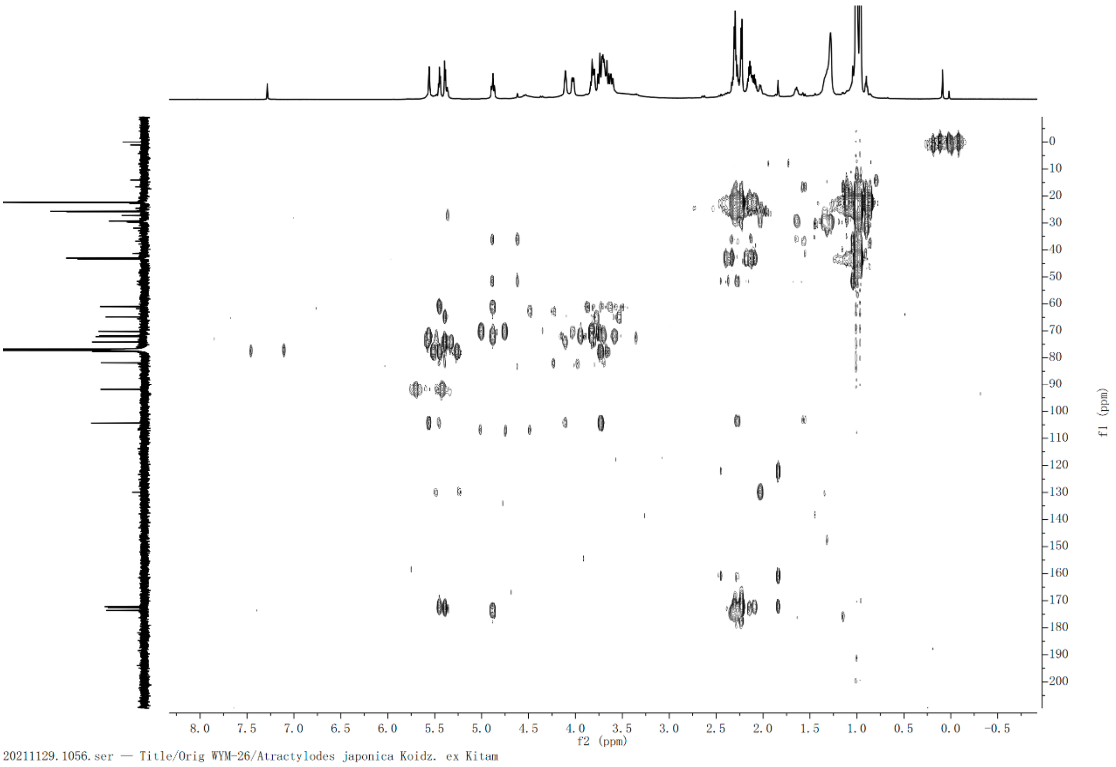

Figure S8: HMBC NMR spectrum of compound **1**

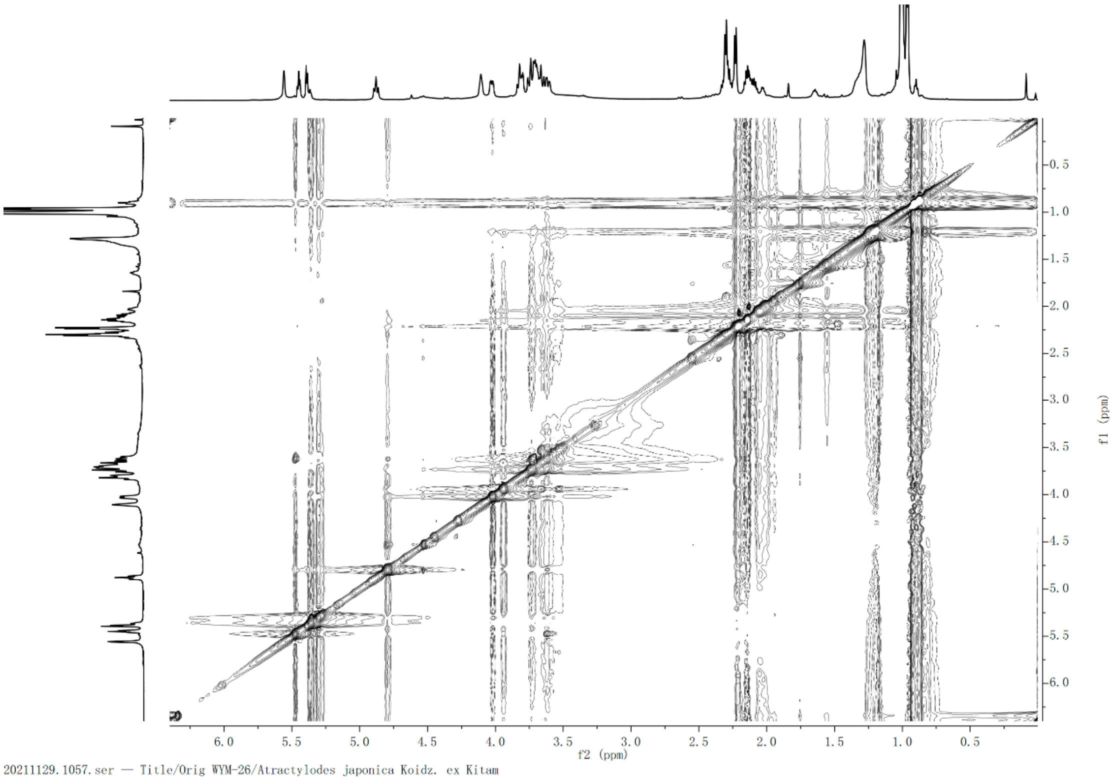

Figure S9: NOESY NMR spectrum of compound **1**

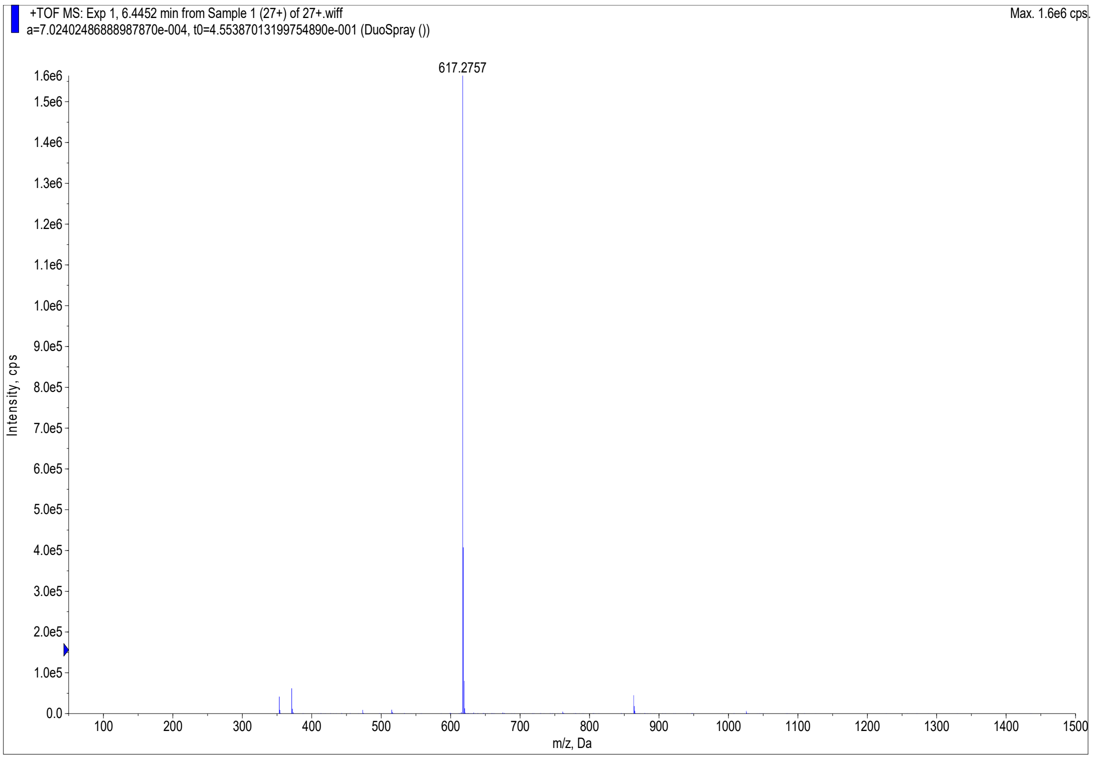

Figure S10: HR-ESI-MS spectrum of compound **2**

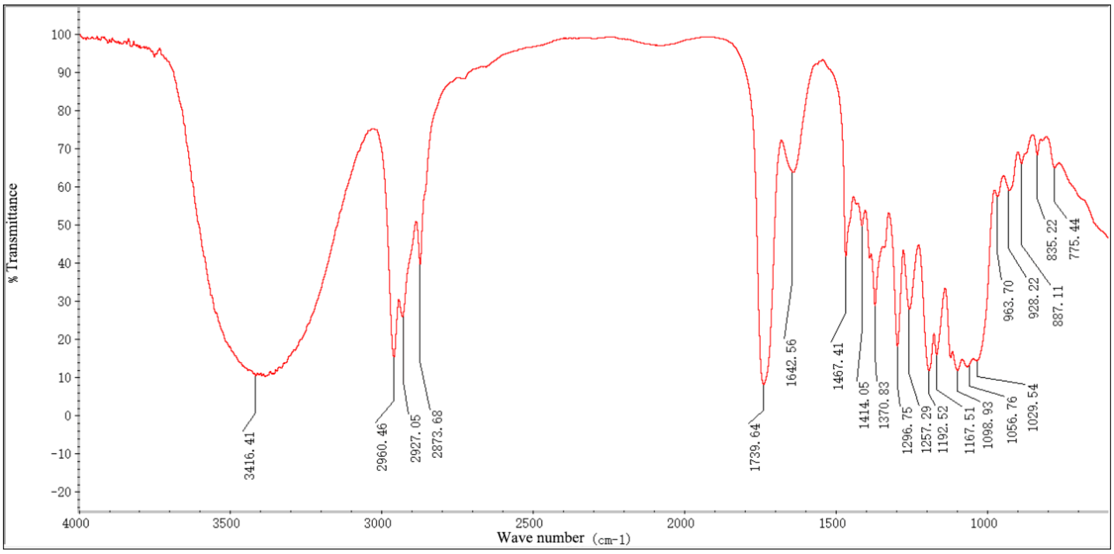

Figure S11: IR spectrum of compound **2**

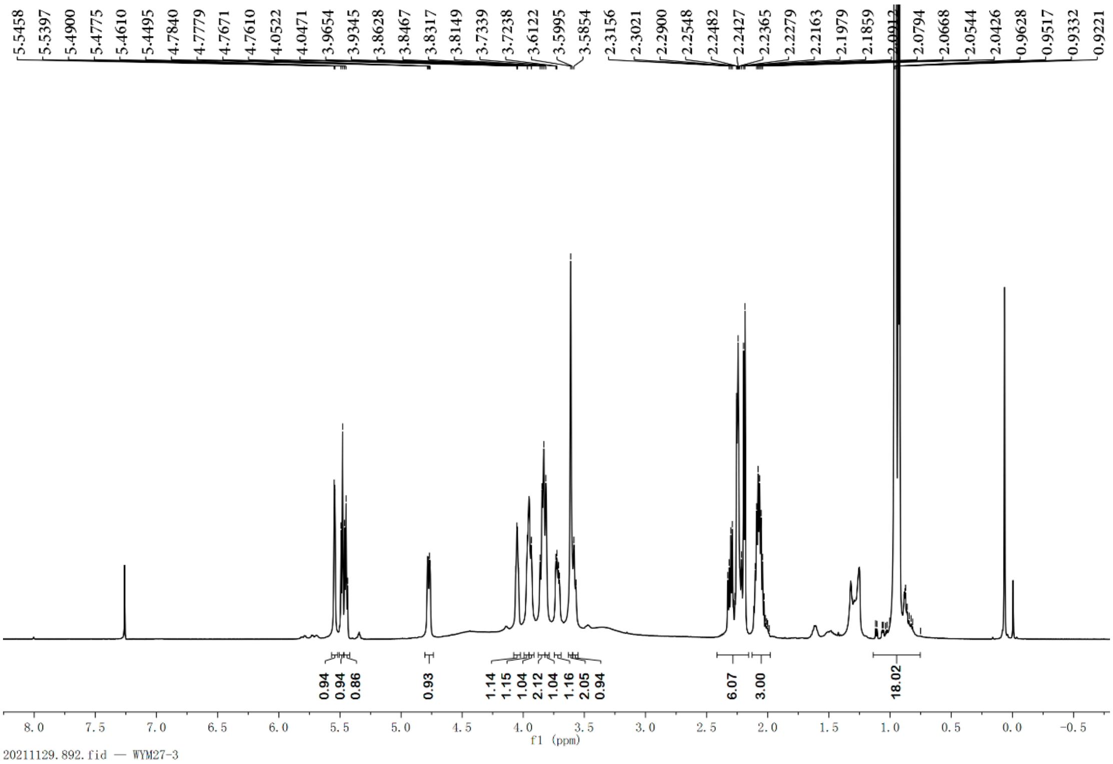

Figure S12: ^{1}H-NMR spectrum of compound **2** (600 MHz, $CDCl_3$)

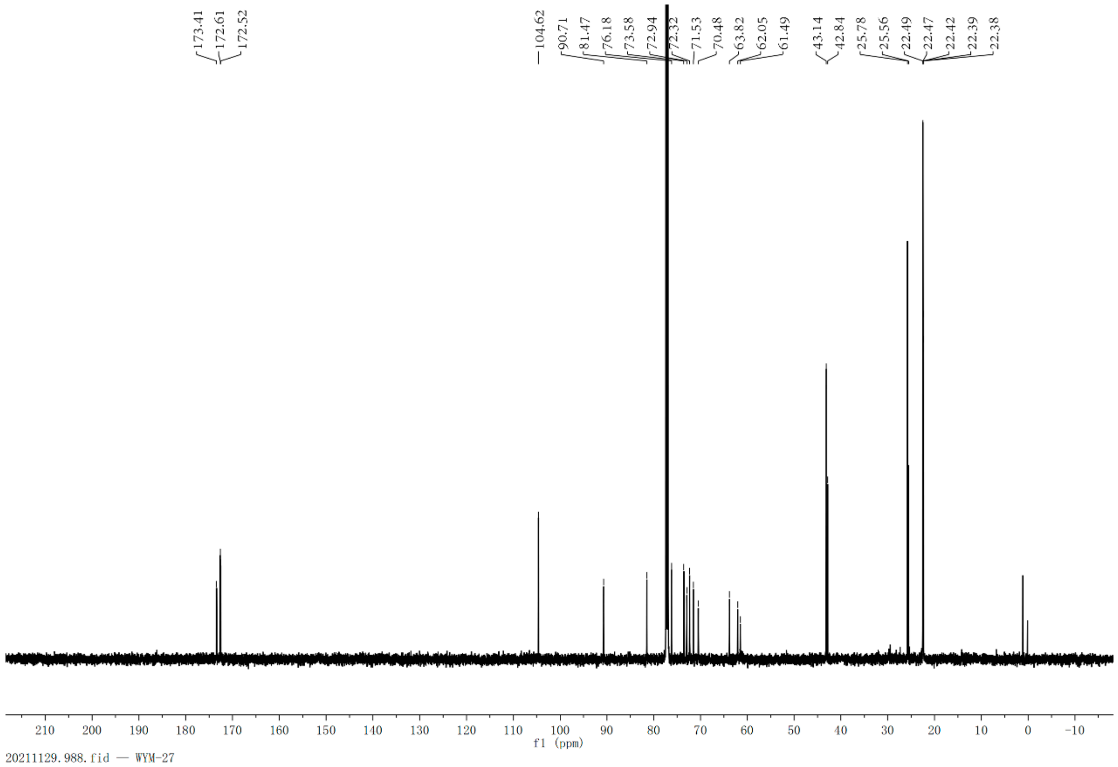

Figure S13: ^{13}C-NMR spectrum of compound **2** (150 MHz, $CDCl_3$)

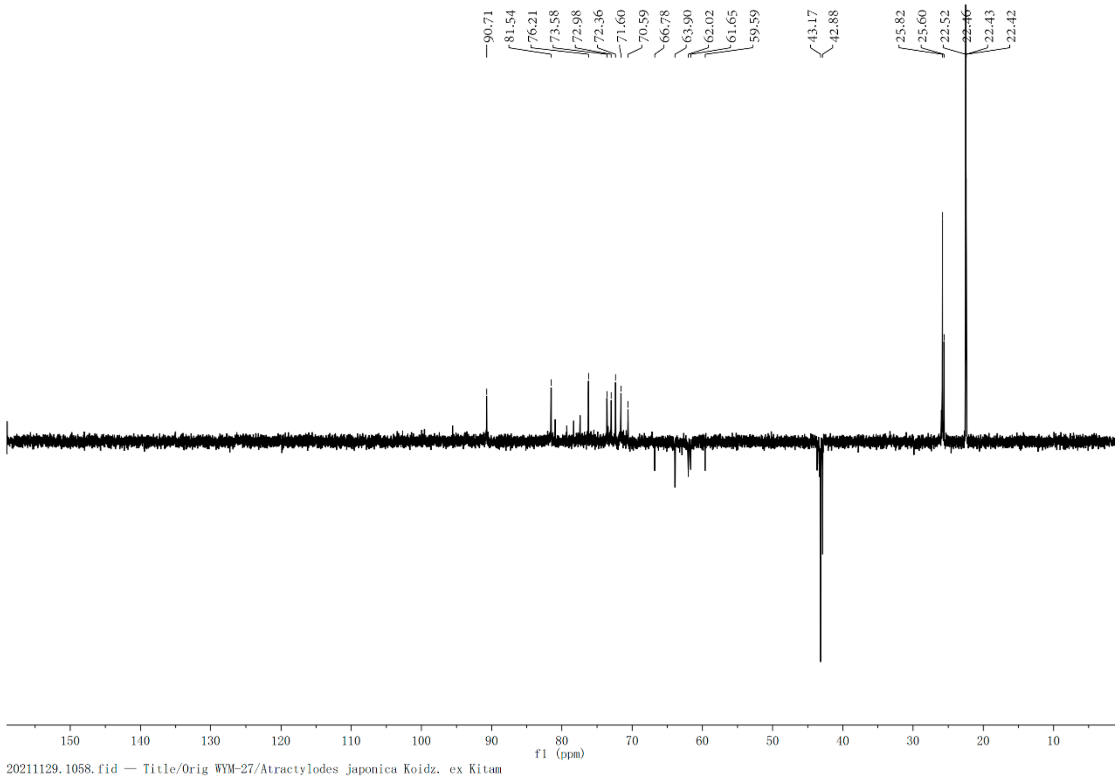

Figure S14: DEPT 135° NMR spectrum of compound **2**

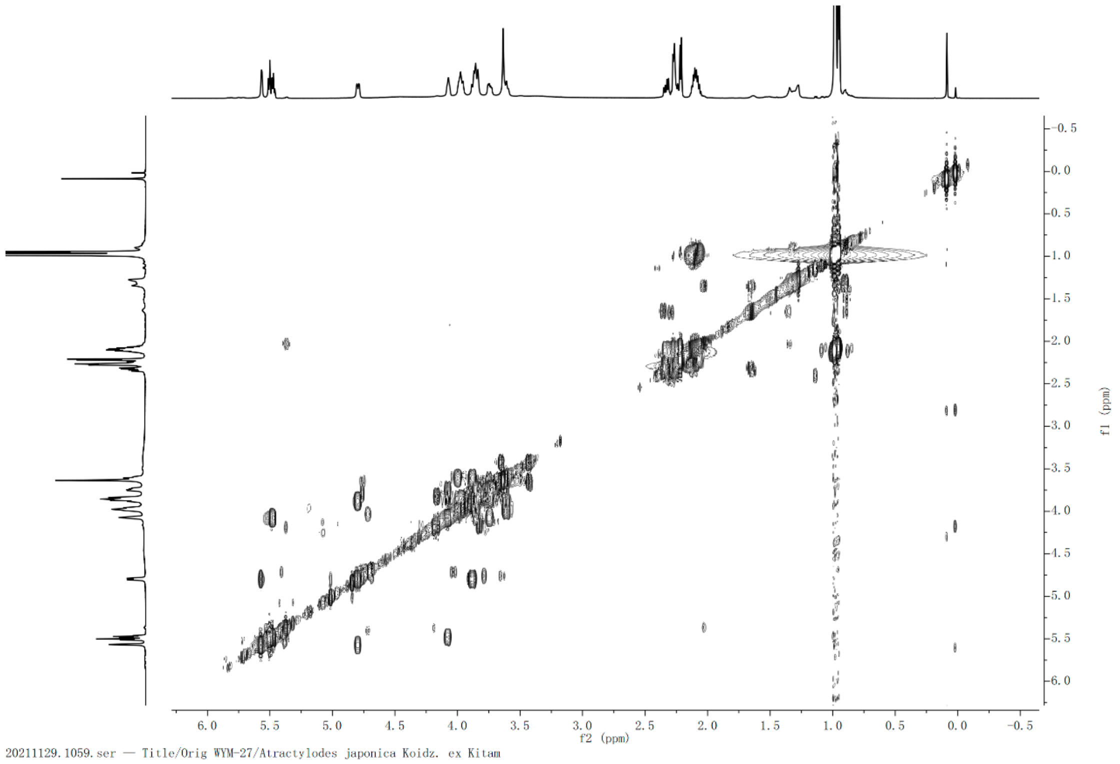

Figure S15: ^{1}H-^{1}H COSY NMR spectrum of compound **2**

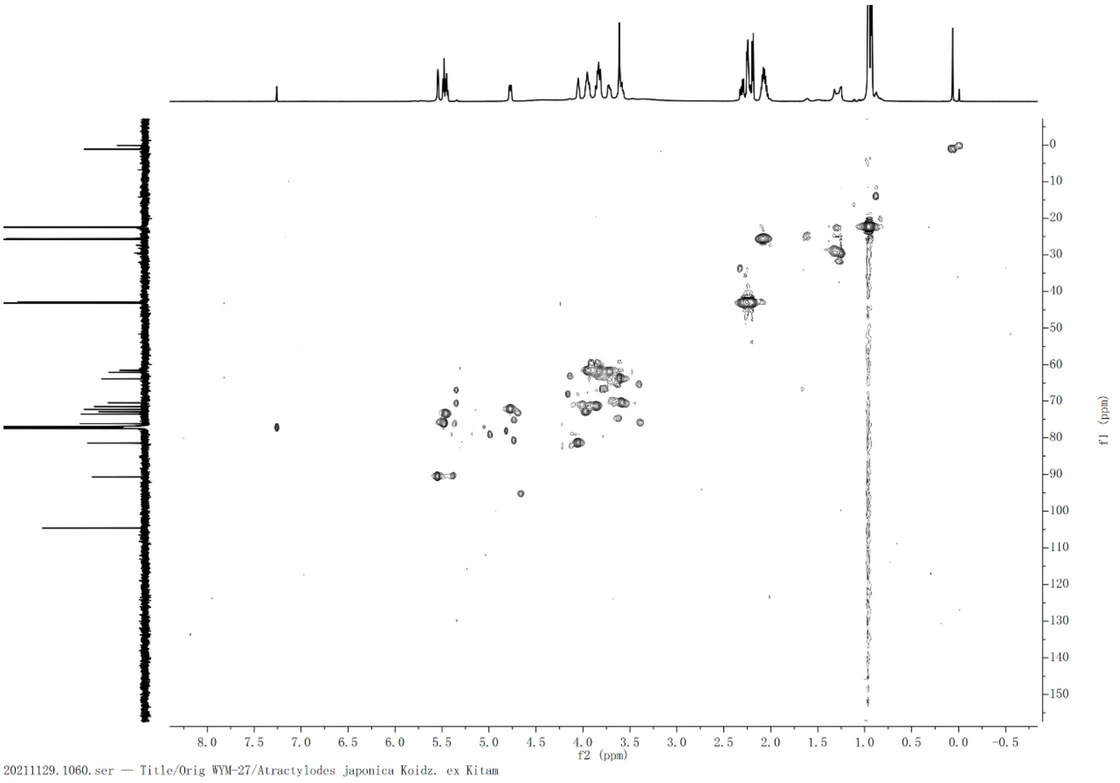

Figure S16: HSQC NMR spectrum of compound **2**

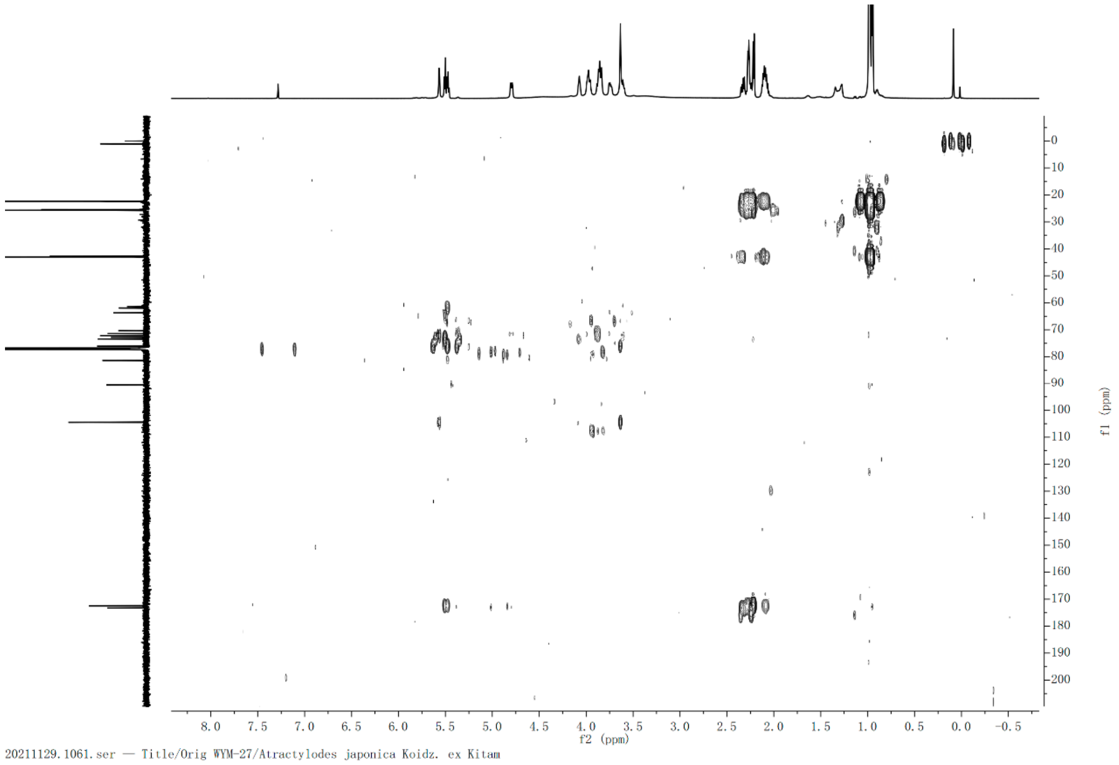

Figure S17: HMBC NMR spectrum of compound **2**

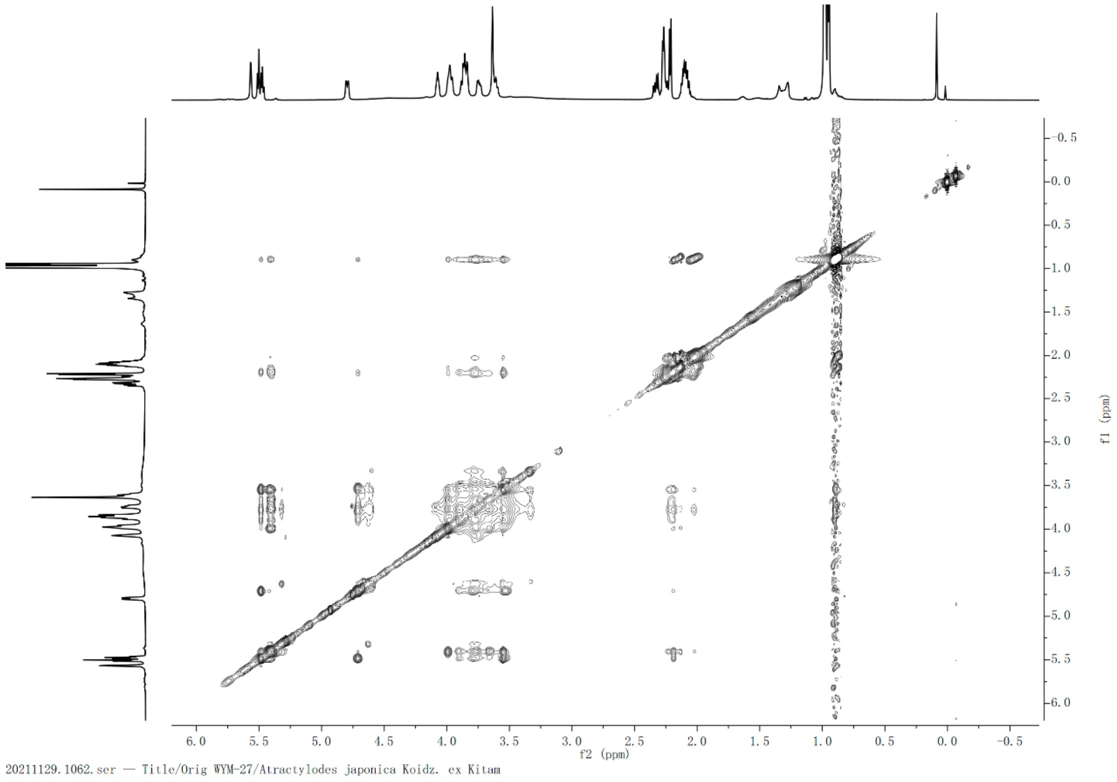

Figure S18: NOESY NMR spectrum of compound **2**

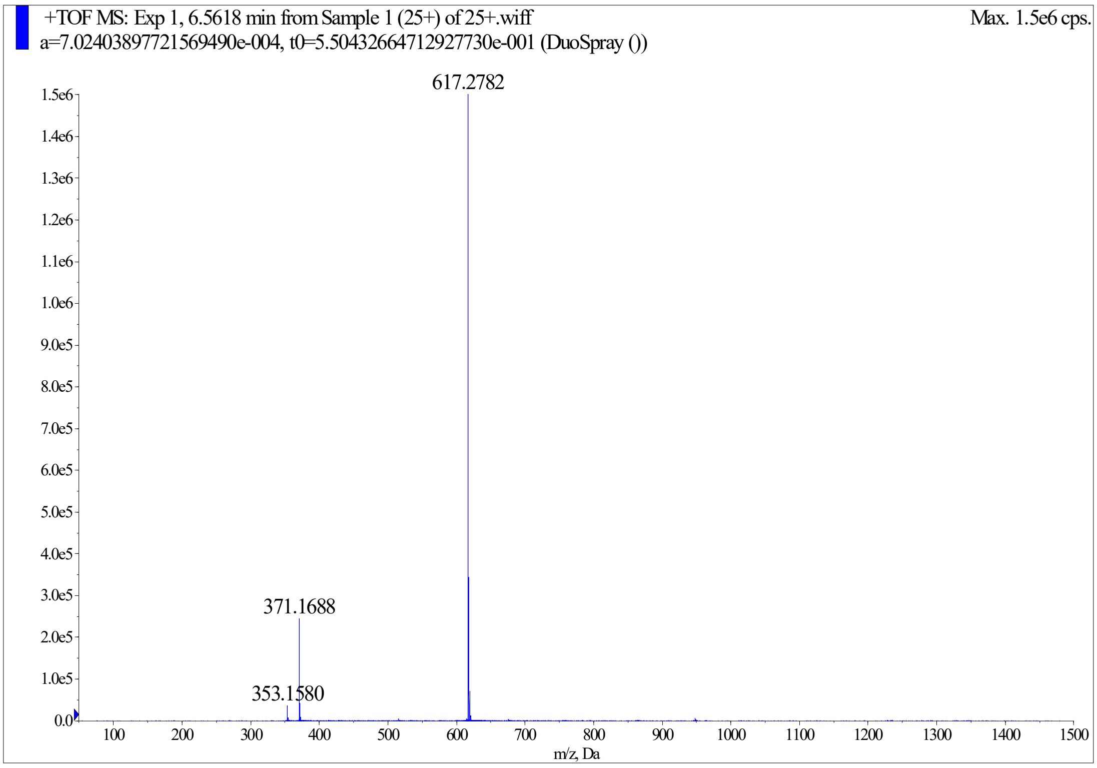

Figure S19: HR-ESI-MS spectrum of compound **3**

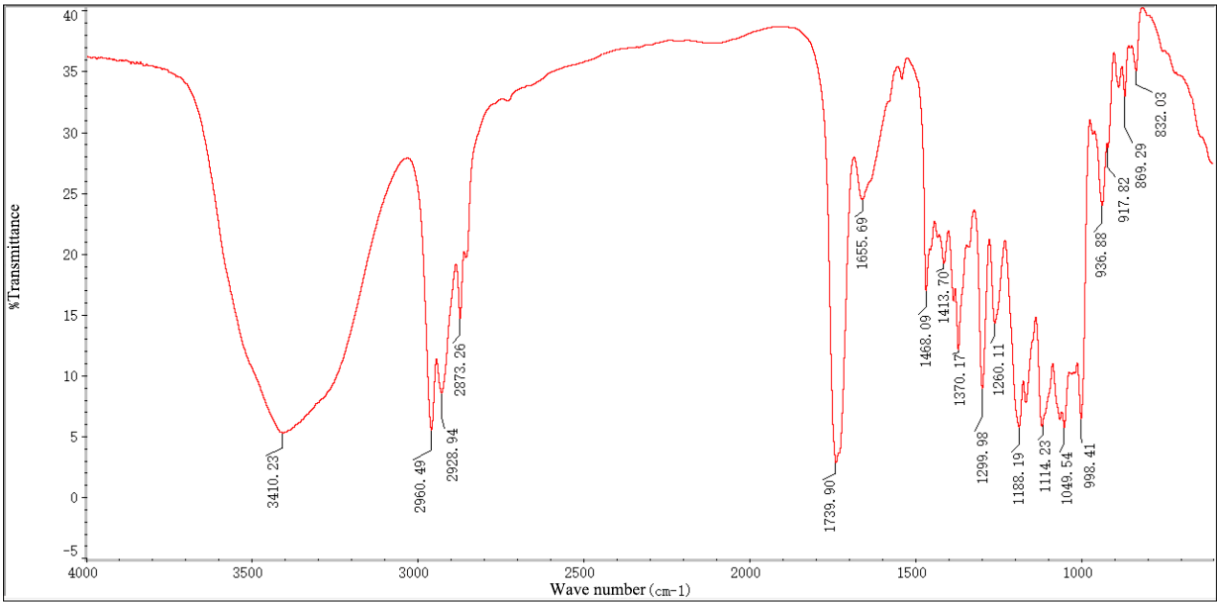

Figure S20: IR spectrum of compound **3**

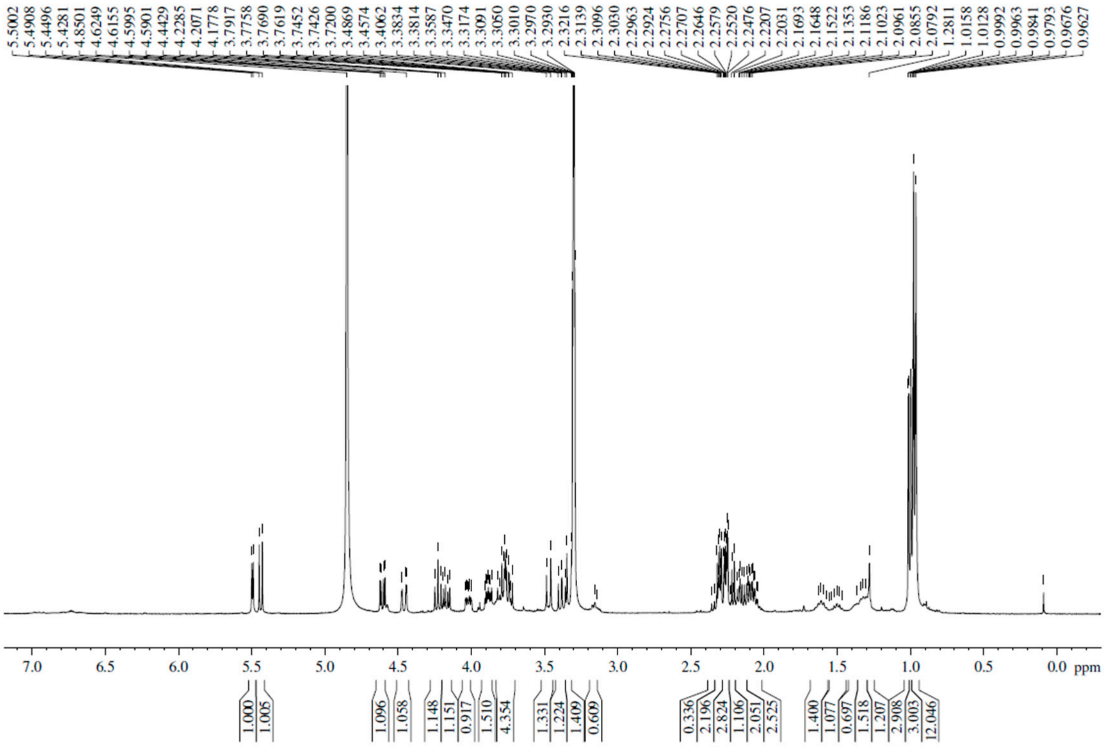

Figure S21:^{1}H-NMR spectrum of compound **3** (400 MHz, CD_3OD)

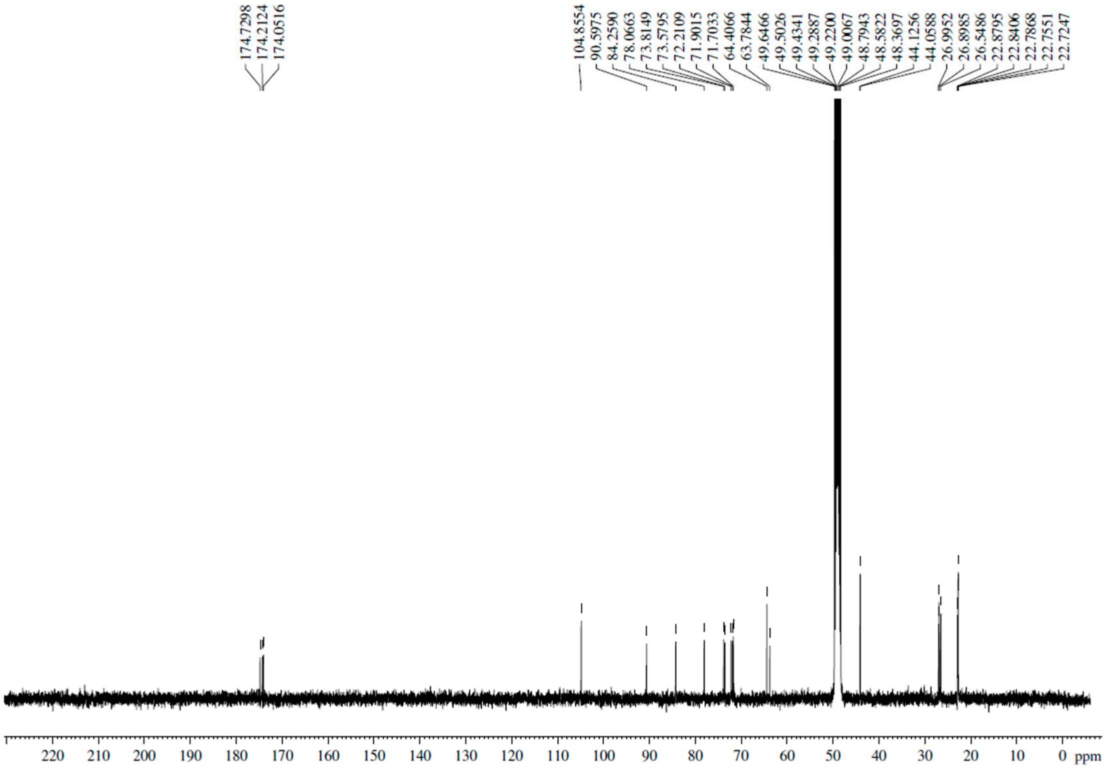

Figure S22:^{13}C-NMR spectrum of compound **3** (150 MHz, CD_3OD)

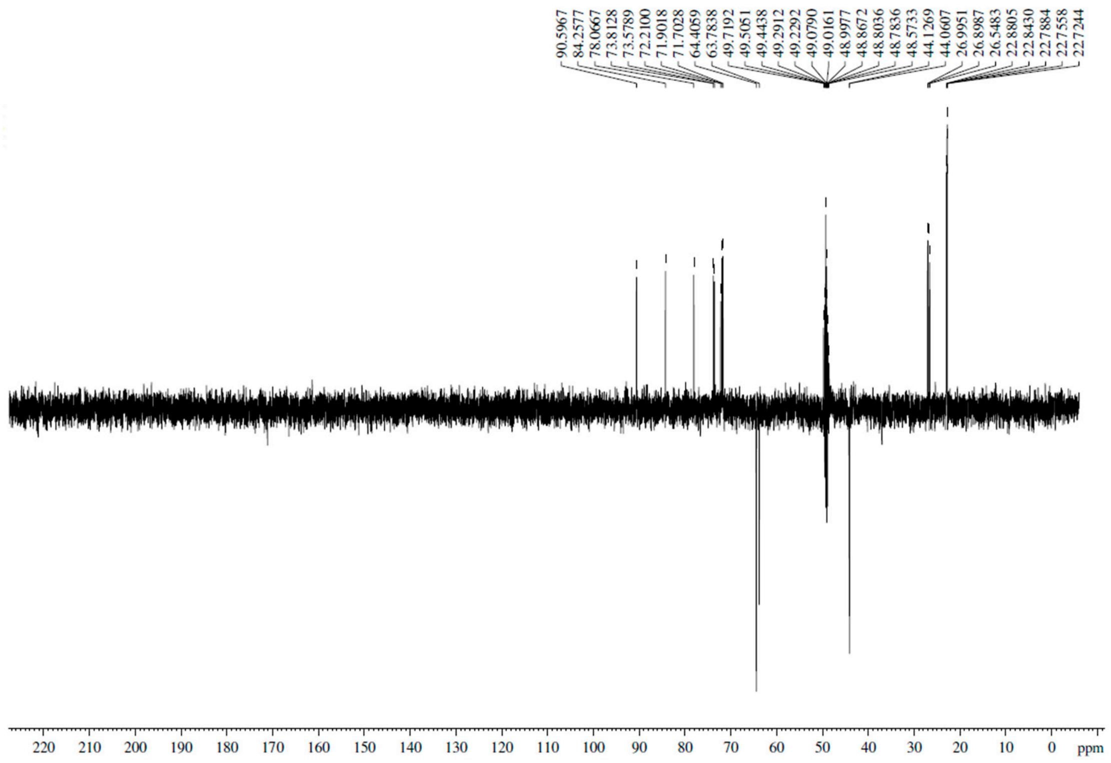

Figure S23: DEPT-135 spectrum of compound **3**

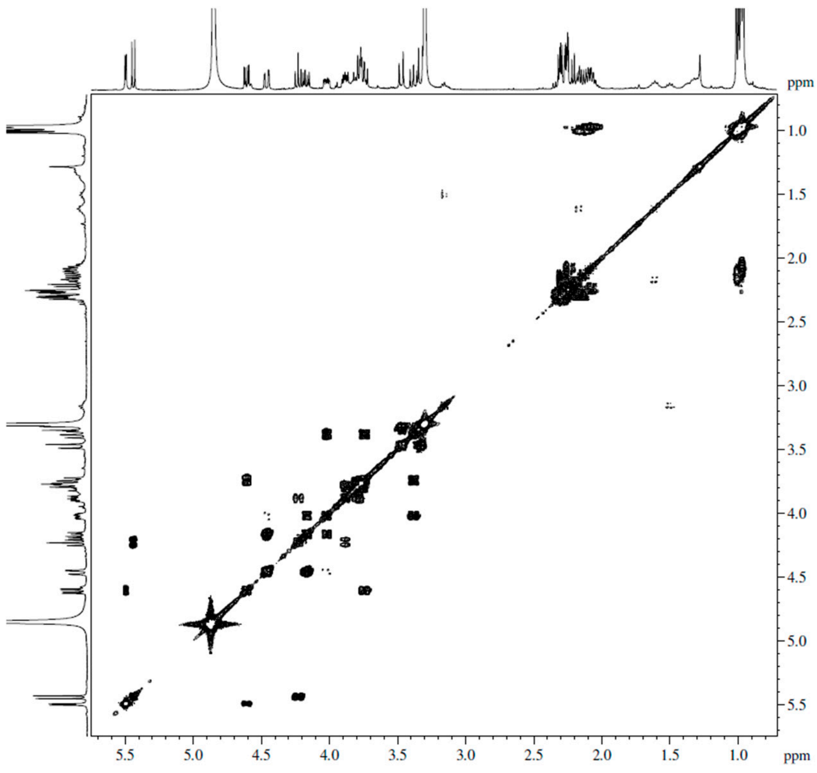

Figure S24: ^{1}H-^{1}H COSY spectrum of compound **3**

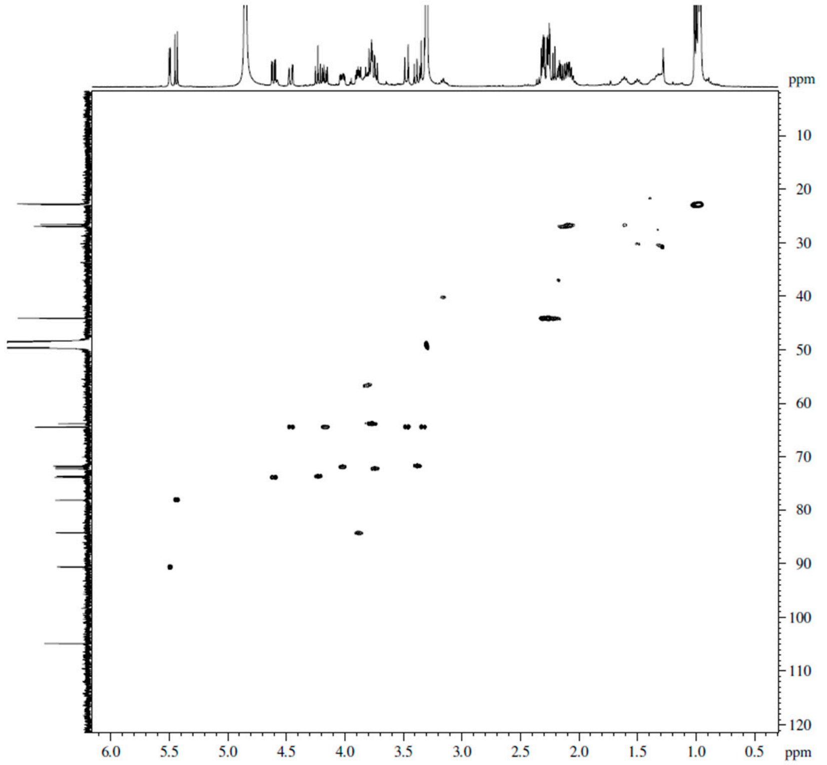

Figure S25: HSQC spectrum of compound **3**

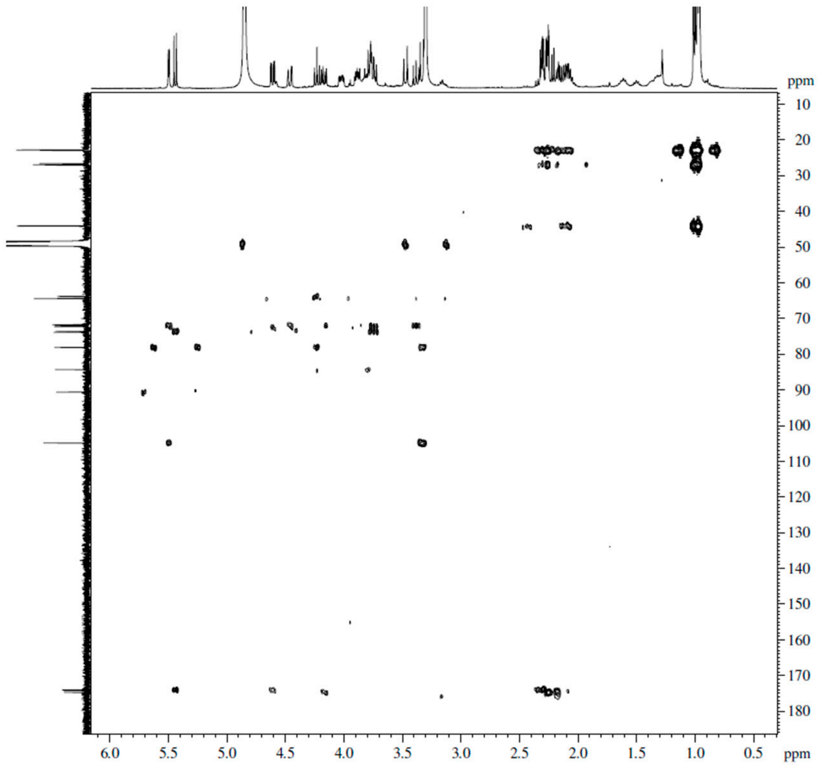

Figure S26: HMBC spectrum of compound **3**

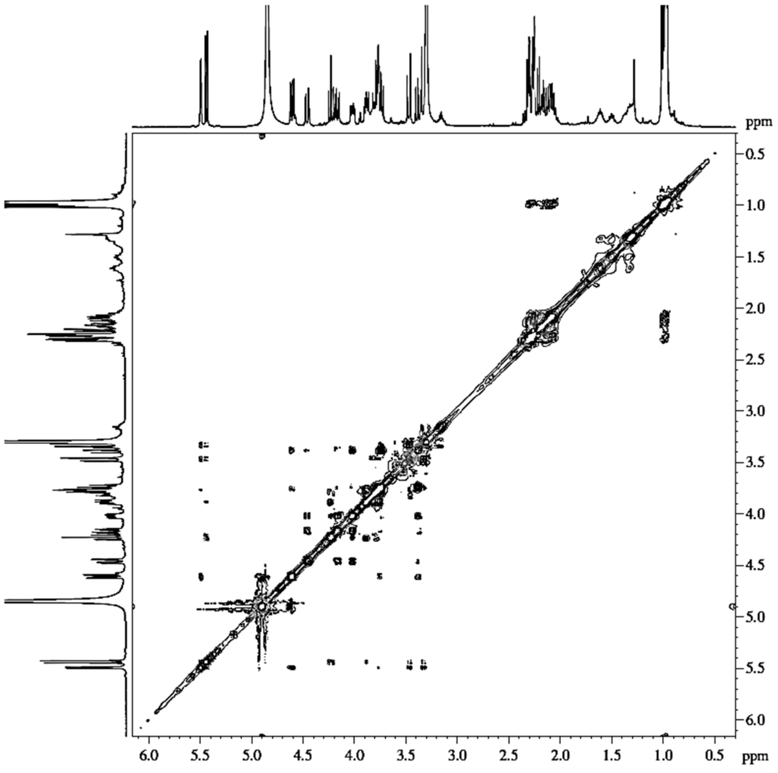

Figure S27: NOESY spectrum of compound **3**

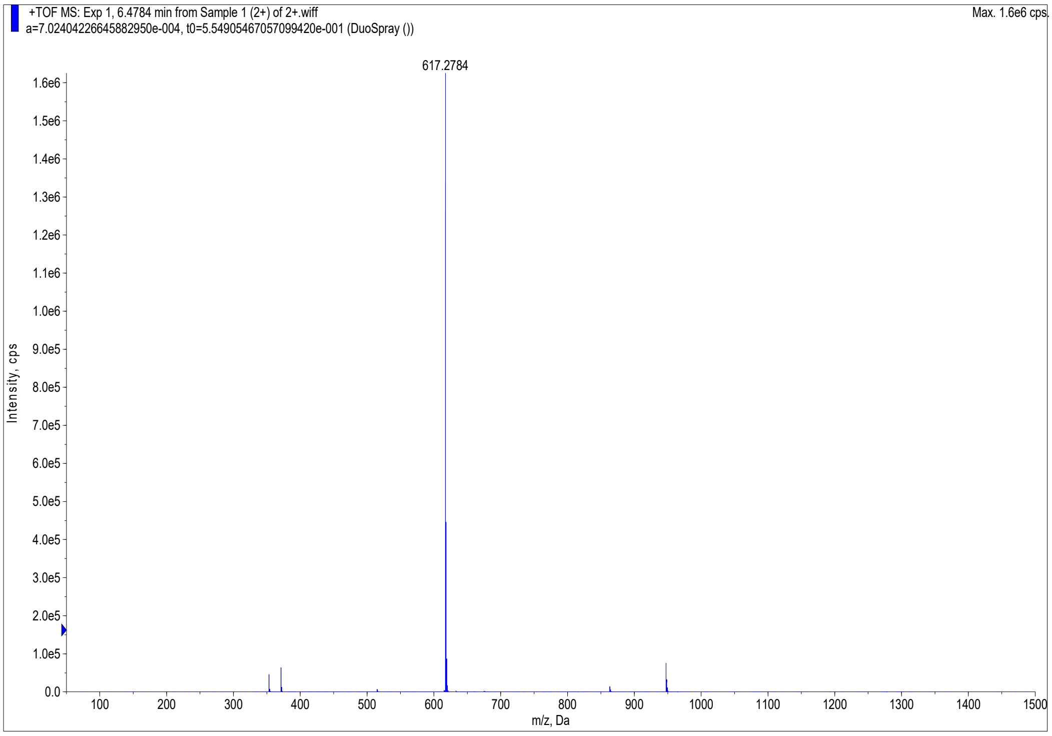

Figure S28: HR-ESI-MS spectrum of compound **4**

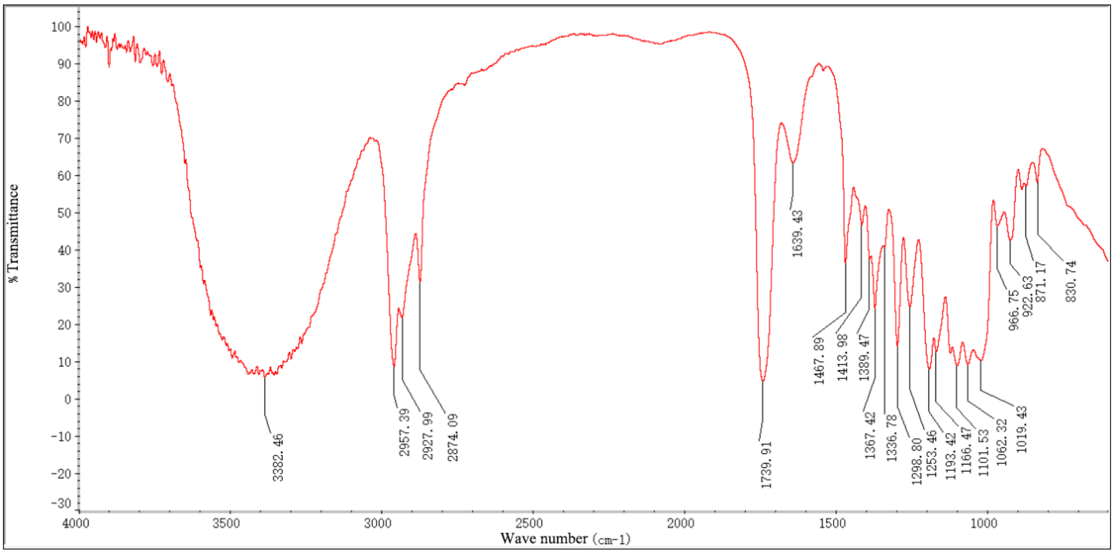

Figure S29: IR spectrum of compound **4**

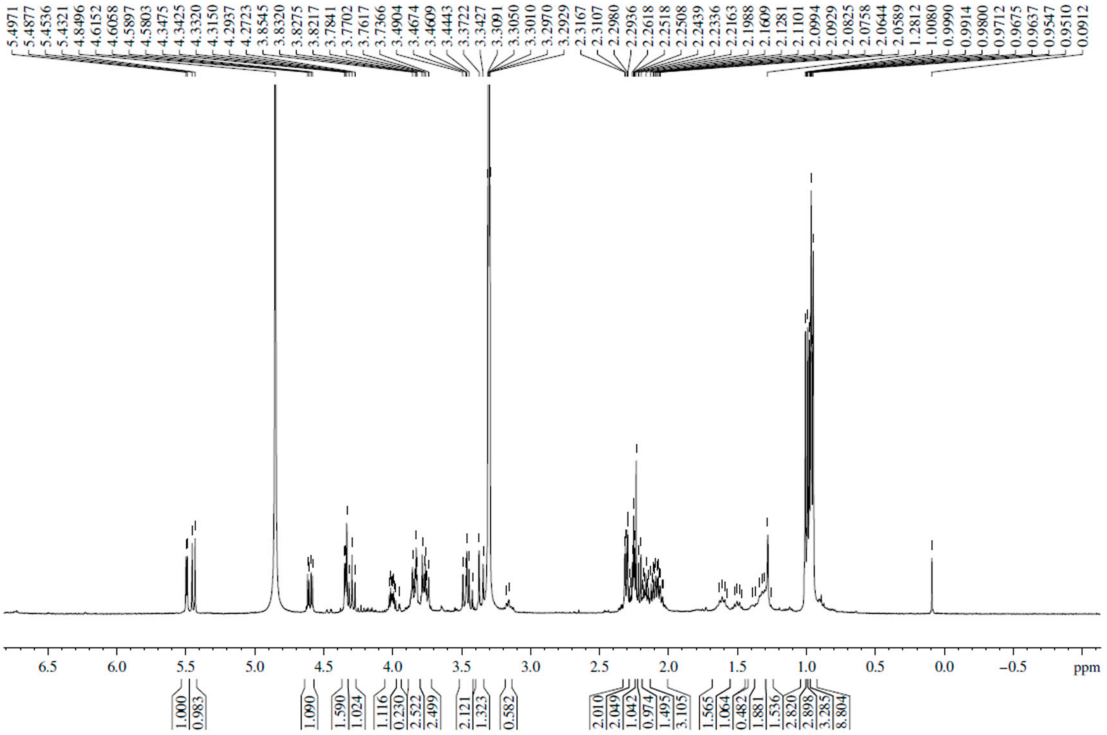

Figure S30: 1H-NMR spectrum of compound **4** (400 MHz, CD_3OD)

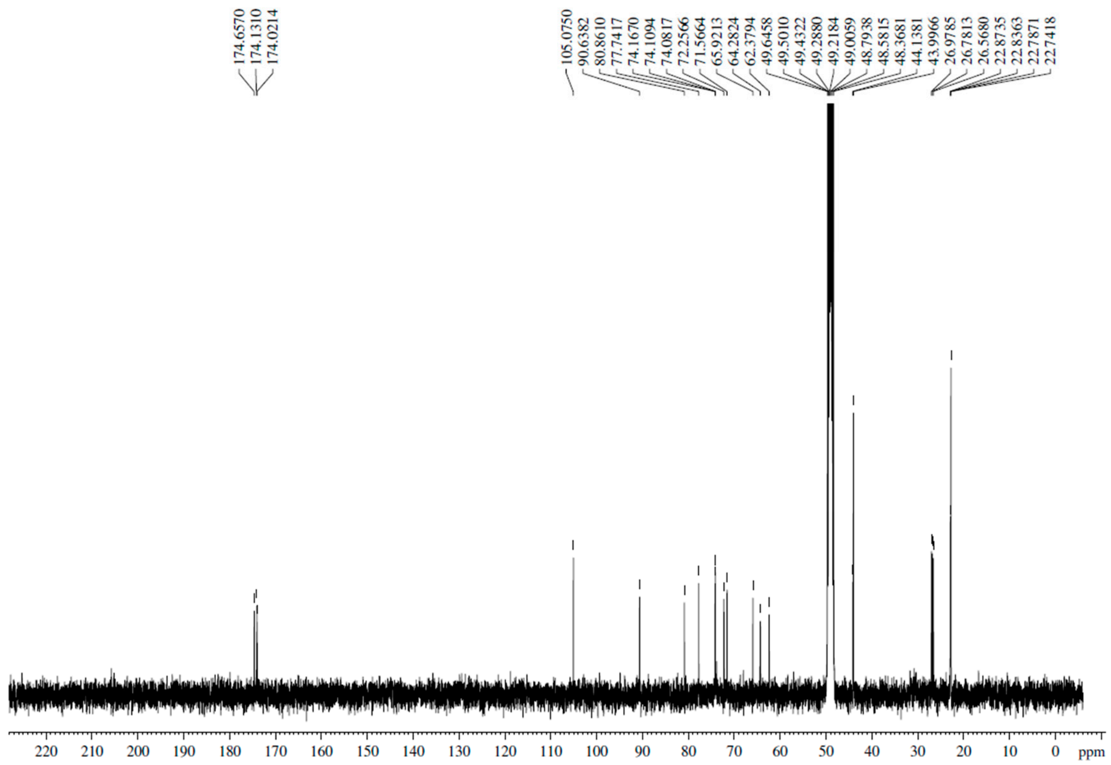

Figure S31: ^{13}C-NMR spectrum of compound **4** (150 MHz, CD_3OD)

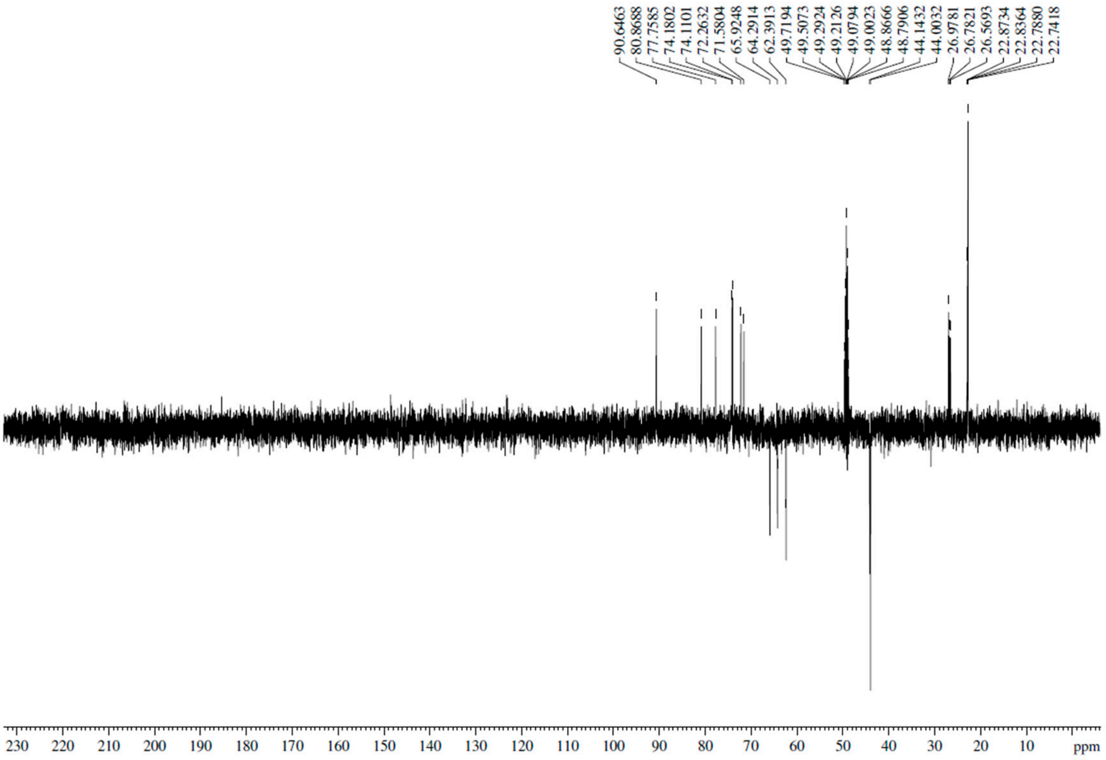

Figure S32: DEPT-135 spectrum of compound **4**

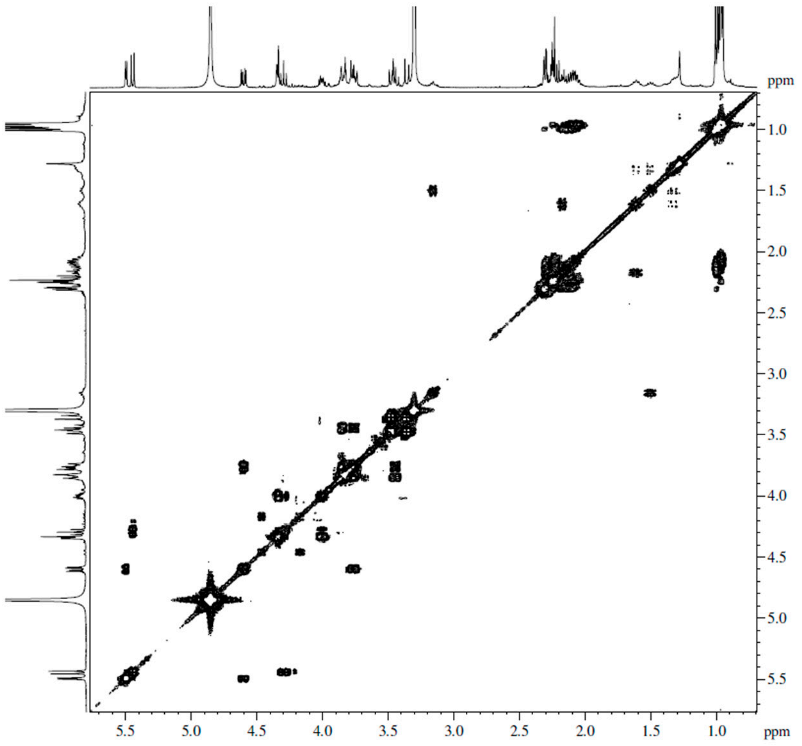

Figure S33: ^{1}H-^{1}H COSY spectrum of compound **4**

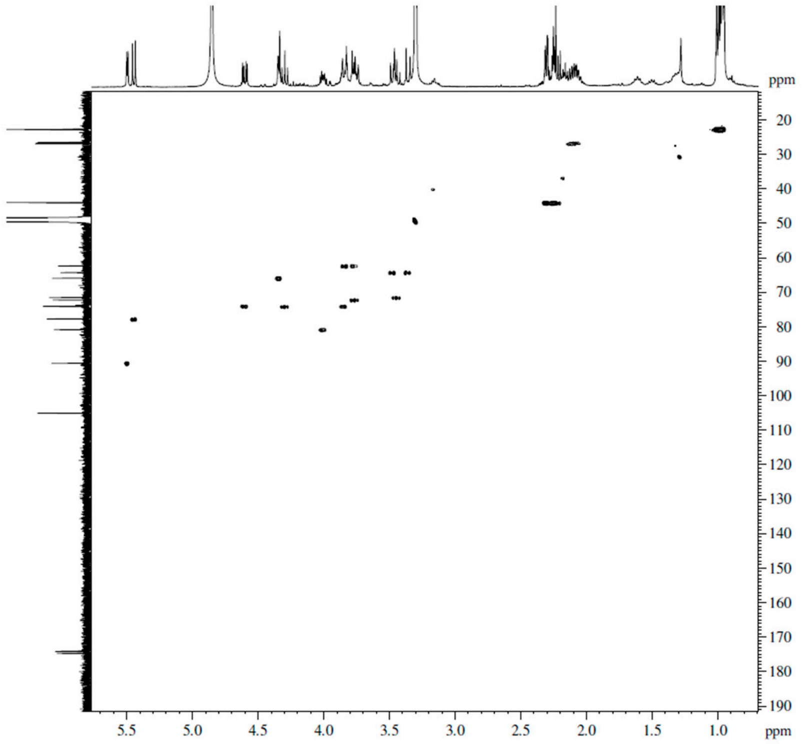

Figure S34: HSQC spectrum of compound **4**

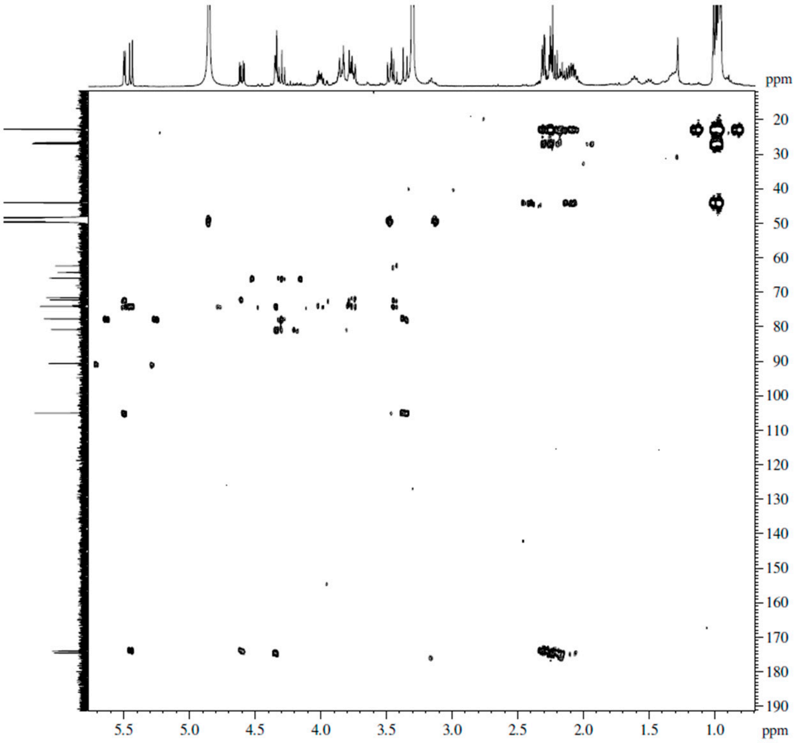

Figure S35: HMBC spectrum of compound **4**

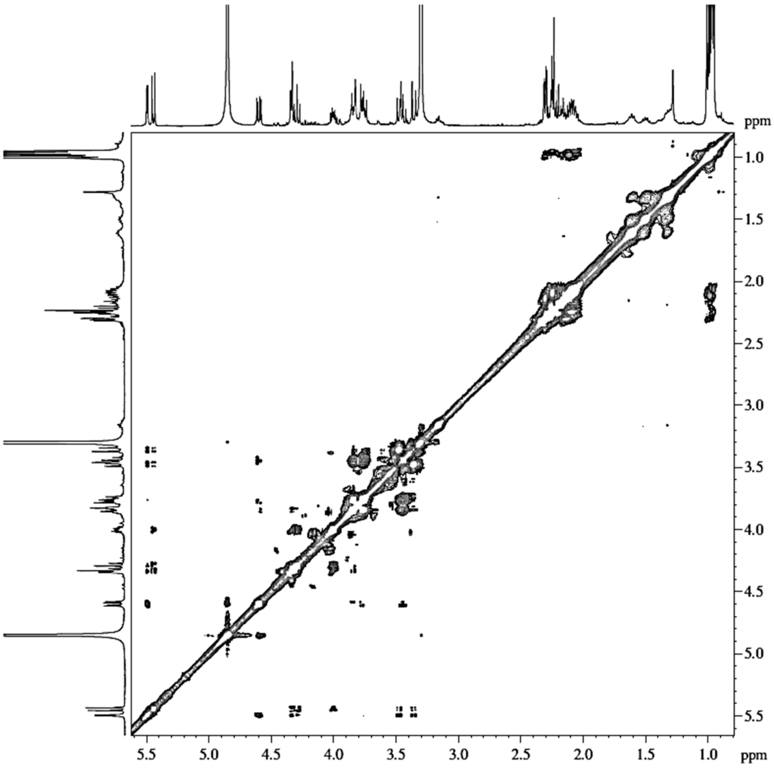

Figure S36: NOESY spectrum of compound **4**

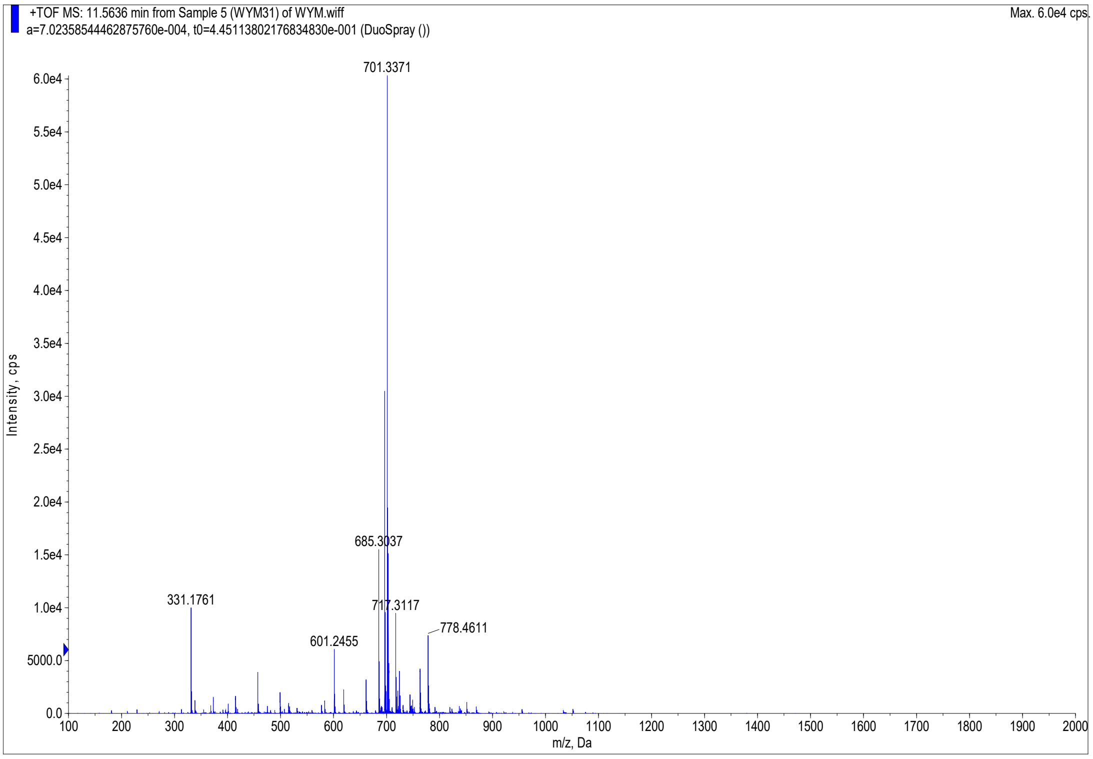

Figure S37: HR-ESI-MS spectrum of compound **5**

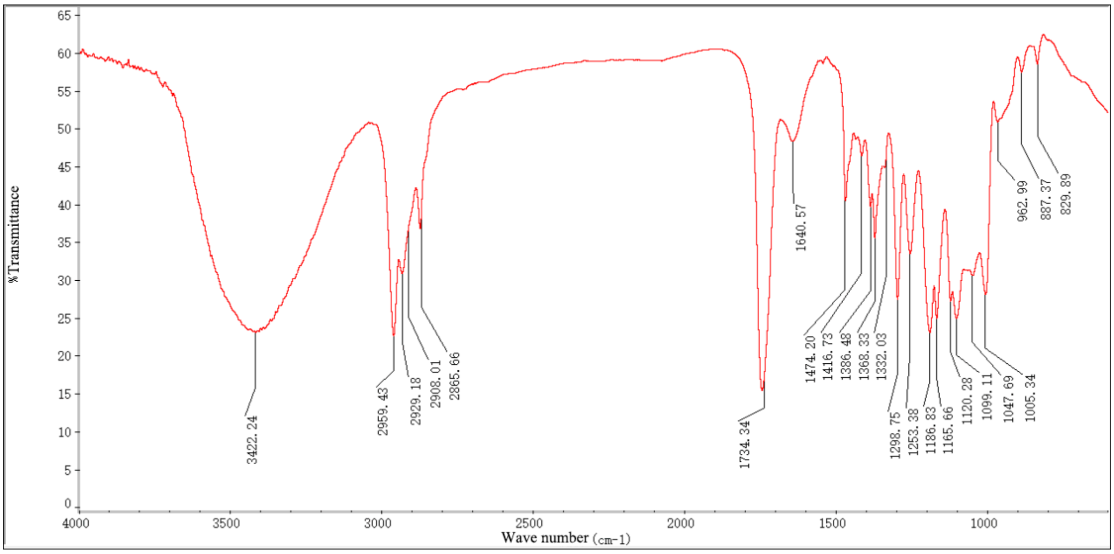

Figure S38: IR spectrum of compound **5**

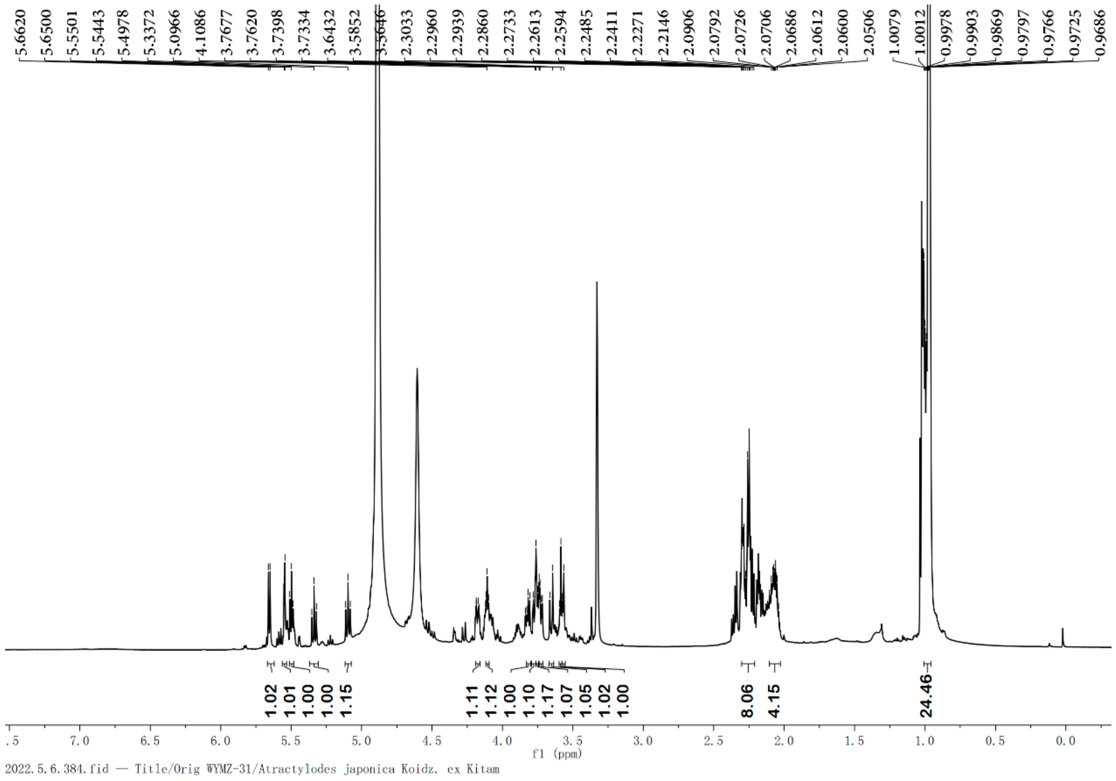

Figure S39: ^{1}H-NMR spectrum of compound **5** (600 MHz, CD_3OD)

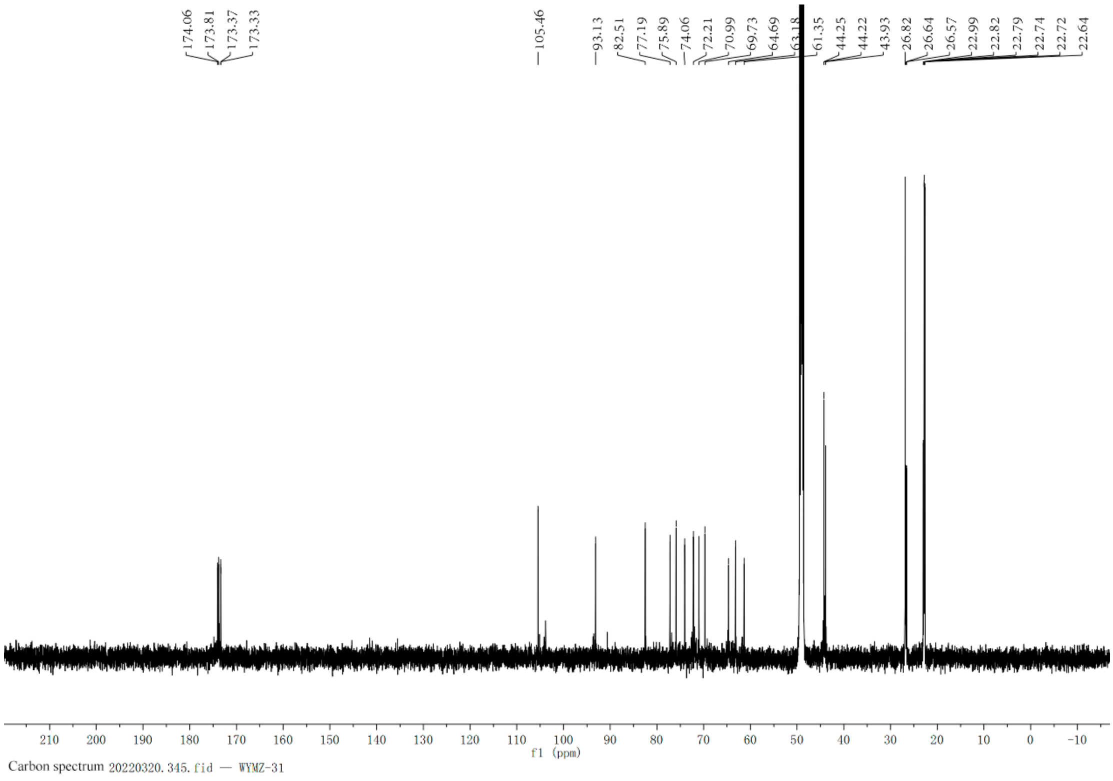

Figure S40: ^{13}C-NMR spectrum of compound **5** (150 MHz, CD_3OD)

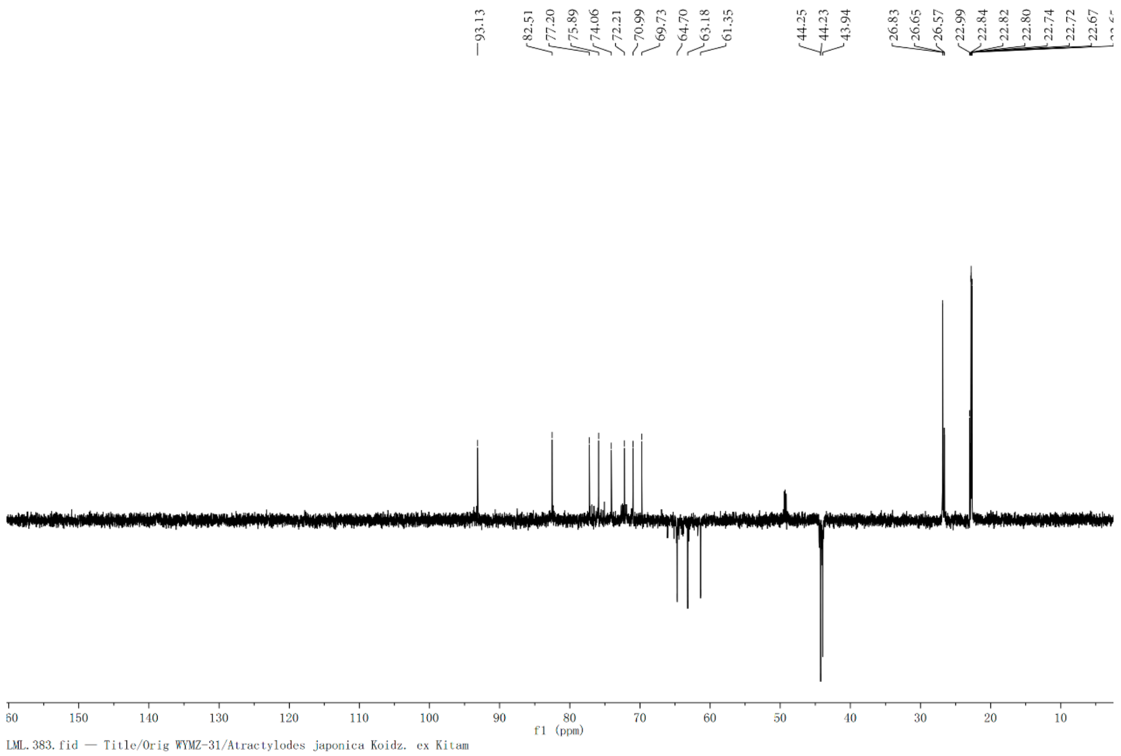

Figure S41: DEPT 135° NMR spectrum of compound **5**

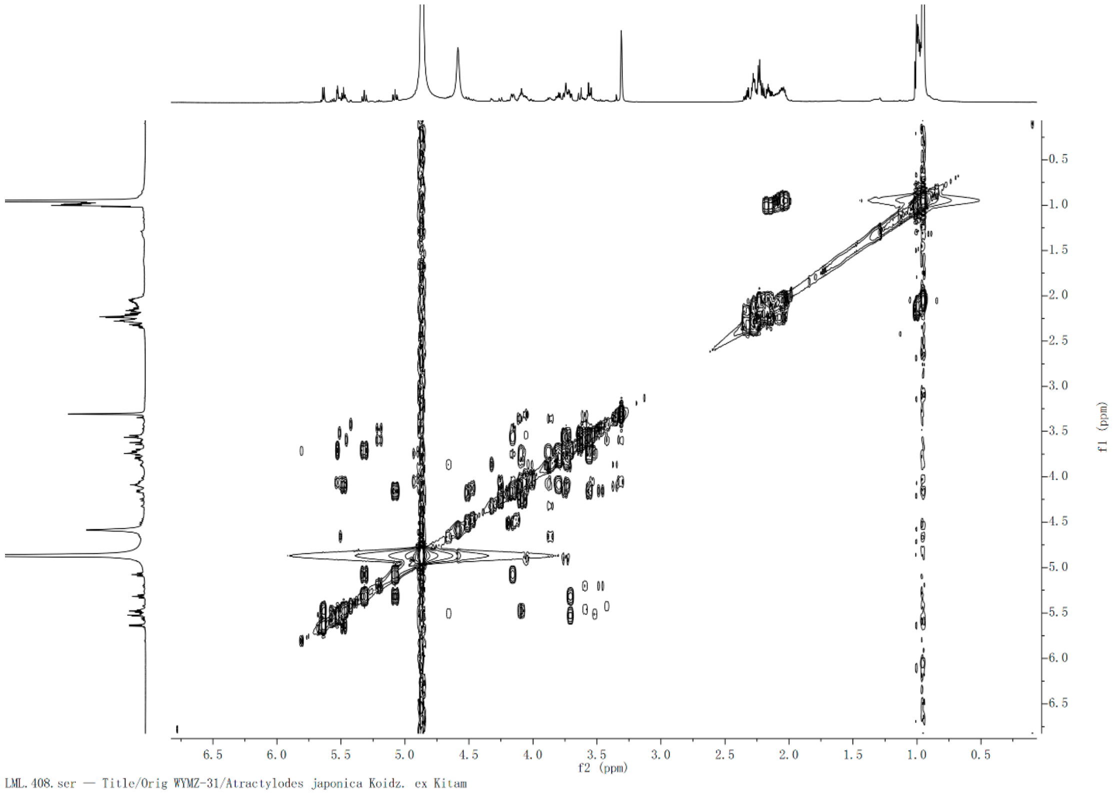

Figure S42: ^{1}H-^{1}H COSY NMR spectrum of compound **5**

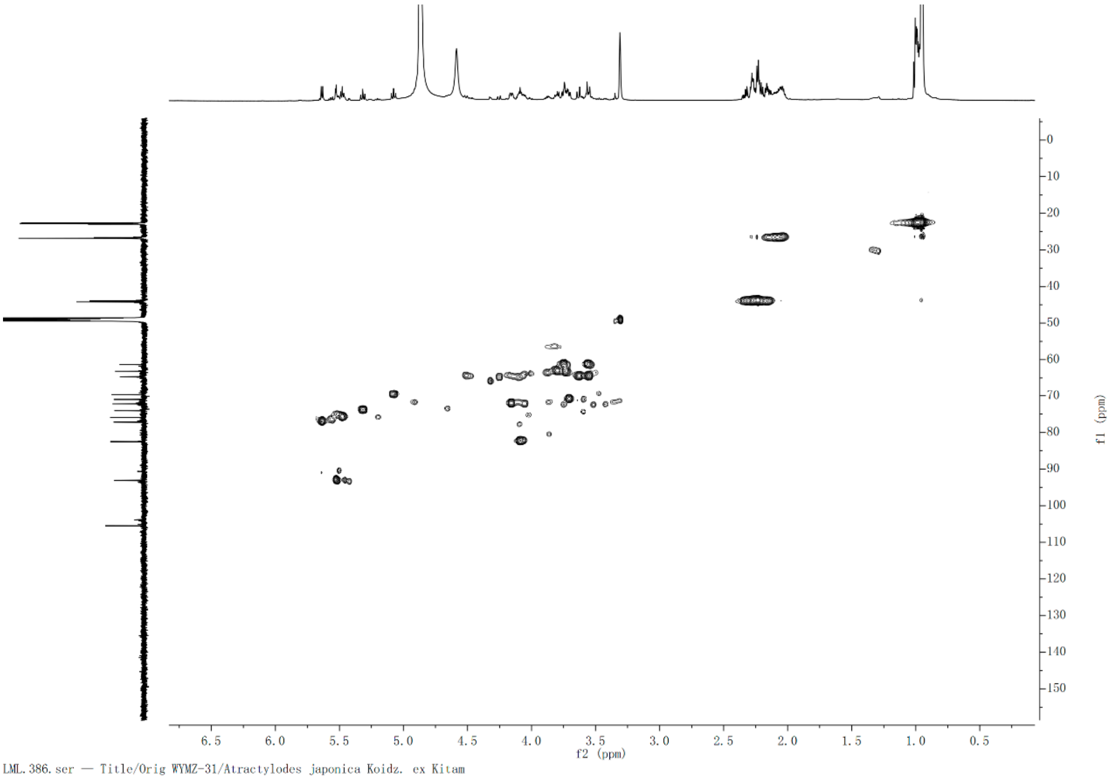

Figure S43: HSQC NMR spectrum of compound **5**

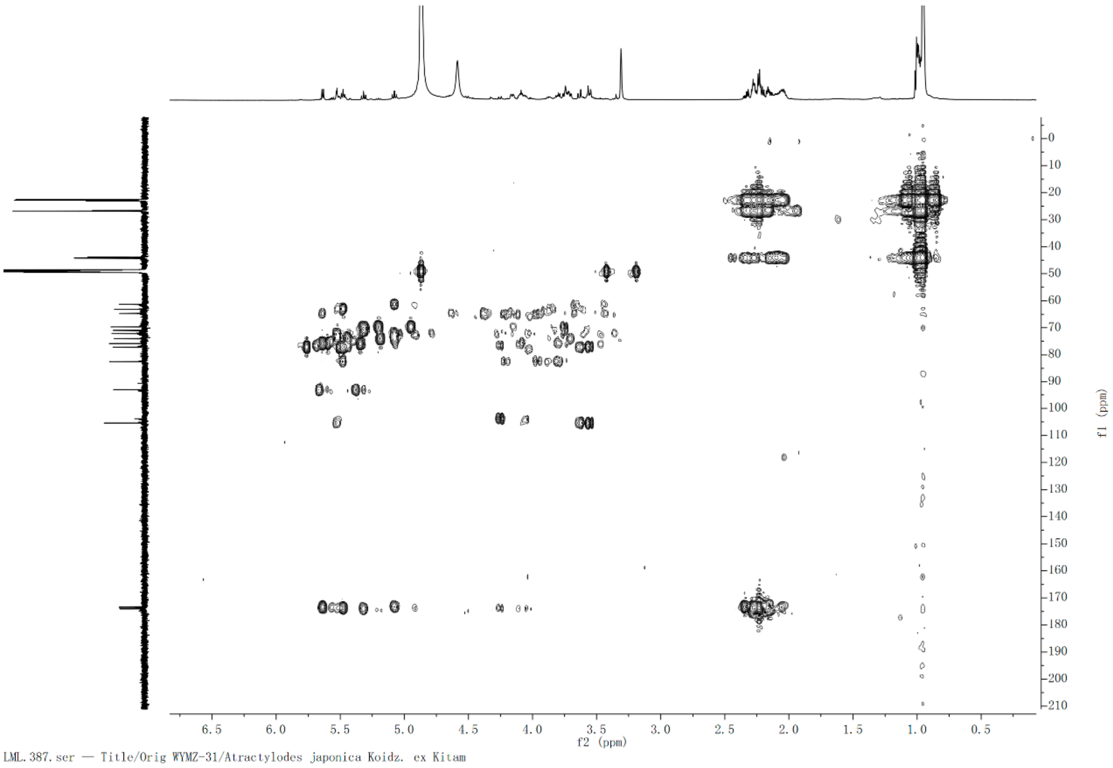

Figure S44: HMBC NMR spectrum of compound **5**

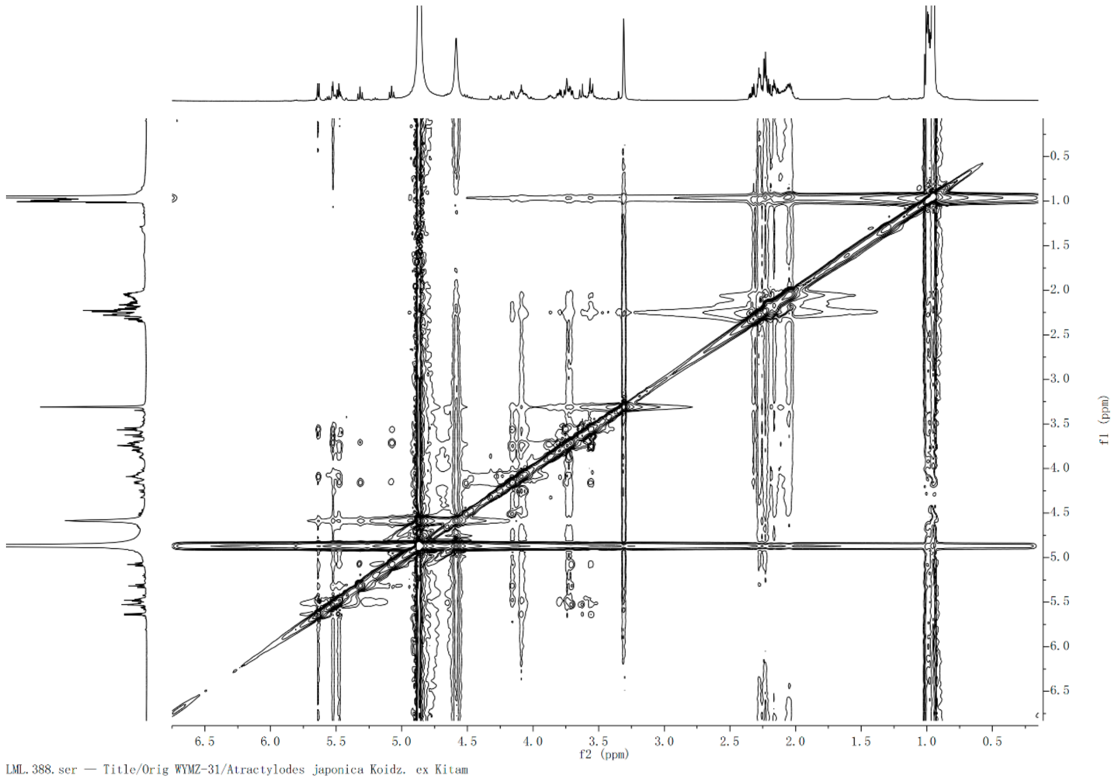

Figure S45: NOESY NMR spectrum of compound **5**

Table S1: ^{13}C-NMR ($CDCl_3$, 150 MHz) spectroscopic data of compounds **6-12** (δ in ppm, J in Hz)

No.	6	7	8	9	10	11	12
1	93.2	93.3	91.0	91.1	90.2	89.7	90.2
2	71.2	72.4	73.7	73.7	73.9	72.5	72.1
3	75.9	76.2	70.1	72.1	72.2	70.0	71.0
4	69.6	74.7	71.9	71.7	71.9	71.2	70.5
5	72.4	72.9	72.1	71.9	73.7	71.3	70.7
6	64.4	64.7	61.7	64.4	64.7	61.7	63.6
1'	64.5	63.8	63.8	64.0	64.5	63.9	62.7
2'	105.0	106.4	105.7	105.8	104.9	104.6	104.8
3'	76.9	77.2	76.3	76.0	77.7	75.4	75.6
4'	75.8	76.1	75.4	75.6	71.9	74.0	74.0
5'	82.3	82.5	82.2	82.4	80.9	78.4	78.6
6'	63.9	63.8	63.4	63.4	65.3	63.9	64.0
1''	174.6	178.9	174.0	174.7	174.7	173.4	174.3
	174.3	174.5	173.7	174.1	174.4	172.9	172.8
	173.4	173.5	173.6	173.6	174.1	172.6	172.7
			173.2	173.4	174.0	172.4	172.5
						172.1	171.9
2''	44.4	440	44.3	44.0	44.1×2	43.3	43.1
	44.0	43.9×2	44.0	43.9	44.0×2	43.0×3	43.0×3
	43.9		43.9	43.8×2		42.9	42.8
			43.8				
3''	26.9	26.8	26.8×2	26.8	27.0	25.8	25.7
	26.8×2	26.9×2	26.7	26.8×2	26.9	25.7	25.6×3
			26.5	26.5	26.8	25.6×3	25.5
					26.6		
4''/5''	22.8×4	22.7×4	22.8×4	22.8×2	22.9×4	22.4×6	22.4×4

	22.7×2	22.6×2	22.7×2<br>22.6×2	22.7×4<br>22.6×2	22.8×4	22.3×4	22.3×6

Table S2: ^{1}H-NMR ($CDCl_3$, 600 MHz) spectroscopic data of compounds **6-12** (δ in ppm, J in Hz)

**No.**	**6**	**7**	**8**	**9**	**10**	**11**	**12**
1	5.45 (d, 3.6)	5.41 (d, 4.0)	5.53 (d, 4.4)	5.63 (d, 3.7)	5.55 (d, 3.8)	5.57 (d, 3.7)	5.52 (d, 3.8)
2	3.58 (dd, 10.0, 3.6)	3.43, (dd, 9.6, 4.0)	4.65 (dd, 10.2, 3.7)	4.74 (dd, 10.2, 3.7)	4.61 (dd, 10.2, 3.8)	4.89 (dd, 9.2, 4.8)	4.80 (dd, 10.1, 3.7)
3	5.19 (t, 9.6)	3.64, (dd, 9.6,8.8)	3.78 m	3.94 (t, 9.8)	3.77 (t, 9.0)	3.97 m	3.99 (t, 9.2)
4	3.45 (t, 9.6)	3.62, (dd, 9.6, 8.8)	3.43 m	4.97 (t, 9.8)	3.40 m	4.87 m	3.37 (t, 9.5)
5	4.16 m	4.06 m	4.10 m	4.11 m	4.34 m	4.11 m	4.22 m
6	3.75 m	4.50 (dd, 12.0, 2.0)	4.51 (dd, 12.0, 1.8)	3.71 m	4.49 (dd, 11.8, 1.9)	3.67 m	4.62 (dd, 12.5, 4.0)
	3.58 m	4.15, (dd, 12.0, 5.6)	4.20 (dd, 12.0, 5.4)	3.57 m	4.20 (dd, 11.8, 5.6)	3.77 m	4.30 (dd, 12.5, 2.1)
1'	3.63 (d, 12.0)	3.56, (d, 12.4	3.84 m	3.51 m	3.49 (d, 11.8)	3.67 m	3.67 m
	3.56 (d, 12.0)	3.54, (d, 12.4	3.54 m	3.46 m	3.38 (d, 11.8)		
3'	5.63, (d, 8.0)	5.60, (d, 7.6	5.70 (d, 8.0)	5.67 (d, 8.0)	5.49 (d, 8.7)	5.52 (d, 8.0)	5.50 (d, 6.9)
4'	5.47, (dd, 8.4, 8.0)	5.43, (dd, 7.6, 7.2)	5.47 (t, 7.9)	5.50 (t, 7.8)	3.40 m	5.50 (d, 8.0)	5.48 m
5'	4.07 m	4.06 m	4.05 m	4.08 m	4.02 m	4.07 m	4.07 m
6'	3.87, (dd, 12.0, 6.8)	3.87, (dd, 12.0, 7.6)	3.82 m	3.73 m	4.33 m	4.47 (dd, 11.8, 7.1)	4.45 (dd, 11.9, 7.5)
	3.72, (dd, 12.0, 3.6)	3.71, (dd, 12.0, 4.0)	3.75 (d, 3.8)	3.49 m		4.36 m	4.33 m
2''	2.31-2.21 m	2.27-2.20 m	2.37 - 2.25 m	2.27 - 2.18 m	2.20-2.32 m	2.19 - 2.29 m	2.19 - 2.29 m
3''	2.15-2.01 m	2.10-2.03 m	2.09 - 2.00 m	2.16 - 2.07 m	2.04-2.18 m	2.03 - 2.16 m	2.03 - 2.14 m
4''/5''	0.98-0.93 m	0.97-0.93 m	0.98 - 0.92 m	1.03 - 0.97 m	0.95-1.01 m	0.93 - 0.99 m	0.93 - 0.98 m
